# Modulation of alveolar macrophage and mitochondrial fitness by medicinal plant-derived nanovesicles to mitigate acute lung injury and viral pneumonia

**DOI:** 10.1186/s12951-024-02473-w

**Published:** 2024-04-18

**Authors:** Lusha Ye, Yanan Gao, Simon Wing Fai Mok, Wucan Liao, Yazhou Wang, Changjiang Chen, Lijun Yang, Junfeng Zhang, Liyun Shi

**Affiliations:** 1 Institute of Translational Medicine, Zhejiang Shuren University, Hangzhou, 310015 Zhejiang China; 2 Department of Immunology and Medical Microbiology, Nanjing University of Chinese Medicine, Nanjing, 210023 China; 3 Department of Medicine, Macau University of Science and Technology, Taipa, Macau China

**Keywords:** Artemisia-derived nanovesicles, Alveolar macrophages, Acute lung injury, Gamma-aminobutyric acid, Mitochondrial function

## Abstract

Acute lung injury (ALI) is generally caused by severe respiratory infection and characterized by overexuberant inflammatory responses and inefficient pathogens-containing, the two major processes wherein alveolar macrophages (AMs) play a central role. Dysfunctional mitochondria have been linked with distorted macrophages and hence lung disorders, but few treatments are currently available to correct these defects. Plant-derive nanovesicles have gained significant attention because of their therapeutic potential, but the targeting cells and the underlying mechanism remain elusive. We herein prepared the nanovesicles from Artemisia annua, a well-known medicinal plant with multiple attributes involving anti-inflammatory, anti-infection, and metabolism-regulating properties. By applying three mice models of acute lung injury caused by bacterial endotoxin, influenza A virus (IAV) and SARS-CoV-2 pseudovirus respectively, we showed that Artemisia-derived nanovesicles (ADNVs) substantially alleviated lung immunopathology and raised the survival rate of challenged mice. Macrophage depletion and adoptive transfer studies confirmed the requirement of AMs for ADNVs effects. We identified that gamma-aminobutyric acid (GABA) enclosed in the vesicles is a major molecular effector mediating the regulatory roles of ADNVs. Specifically, GABA acts on macrophages through GABA receptors, promoting mitochondrial gene programming and bioenergy generation, reducing oxidative stress and inflammatory signals, thereby enhancing the adaptability of AMs to inflammation resolution. Collectively, this study identifies a promising nanotherapeutics for alleviating lung pathology, and elucidates a mechanism whereby the canonical neurotransmitter modifies AMs and mitochondria to resume tissue homeostasis, which may have broader implications for treating critical pulmonary diseases such as COVID-19.

**Supplementary Information:**

The online version contains supplementary material available at 10.1186/s12951-024-02473-w.

## Introduction

Acute lung injury (ALI) is a debilitating disease characterized by aberrant immune cells activation and exuberant proinflammatory cytokines generation, leading eventually to extensive lung tissue damages that culminate at the development of acute respiratory distress syndrome (ARDS) [1, 2]. A variety of pathogens, such as Gram-negative bacteria, influenza A virus (IAV) or SARS-CoV-2, have been demonstrate to cause such severe lung inflammation and injury and yield high morbidity and mortality in critical cases. It has been recognized that deregulated immune cells and uncontrolled inflammatory responses, contribute substantially to disease aggravation and increased mortality. Since the recovery of individuals with severe lung disease is largely determined by successful control of inflammation rather than pathogen eradication [3, 4], novel treatments aimed to alleviate lung damages by targeting host immune systems are in pressing need.

Alveolar macrophages (AMs) are a lung resident macrophage population predominating the alveolar space in resting conditions, exerting an important role in maintaining tissue homeostasis, clearing pathogens and coordinating the innate immune responses in lungs [5, 6]. For instances, AMs can directly eliminate the invading pathogens through the release of a network of cytokines, chemokines and anti-microbial factors. They can also recruit peripheral immune cells to the affected sites to synergistically mediate the innate immune response, or even trigger cellular death pathways to prevent pathogens dissemination [7]. On the other hand, AMs exert the important roles in coordinating the innate immune and inflammatory responses, mediating the regenerative response, and maintaining lung tissue integrity. Owing to their versatile activities, depletion of AM pool either by clodronate administration or pathogenic infections has been demonstrated to cause exaggerate lung pathology and lethal viral pneumonia [8]. Replacement of AMs by monocyte-derived macrophages (MMs) or the shift of AMs from anti-inflammatory M2 to pro-inflammatory M1 type have been characterized as a core signature of severe ALI and ARDS associated with critical respiratory diseases such as COVID-19 [9, 10]. Accordingly, pharmacological interventions aimed to resume AMs fitness or reverse their pathogenic transition in diseased lungs are increasingly recognized as a promising alternative to deal with critical lung inflammatory diseases.

The homeostasis and function of AMs is governed by multiple factors, among which well-organized mitochondria plays a pivotal role in regulating macrophage activity and hence immune homeostasis. Mitochondrial status affects various aspects of AMs including cellular phenotypes, functions, survival, and self-renewal, which are frequently disturbed or impaired during pulmonary illness [11]. Mitochondria, as an evolutionarily conserved and crucial organelle, mediate a wide range of important biological functions, e.g., generation of cellular energy by producing adenosine triphosphate (ATP), maintenance of the redox/oxidative balance, and facilitation of tricarboxylic acid (TCA) cycle to generate the metabolic intermediates for biosynthesis or epigenetic machinery to modify gene expression. Concurrently, mitochondria are signaling hubs controlling cellular apoptosis, metabolic programs, anti-viral immunity and inflammatory responses. Therefore, maintaining mitochondrial integrity is critical for sustenance and functioning of lung resident macrophages, whereas distorted mitochondria may cause energetic exhaustion, oxidative stress and inflammatory responses leading to AMs depletion and immune imbalance that underpin critical pulmonary diseases [12, 13]. To rectify this defect, recent attempts of “mitochondrial transfer” or “mitochondria replacement” have demonstrated promising effects [14, 15]. However, the broader application of such approach is impeded due to several factors, such as limited mitochondria sources, complicated preparing protocols, and inefficient mitochondria delivery, calling for more accessible and feasible means to boost mitochondria integrity and hence AMs fitness for lessened lung pathologies [16, 17].

Recently, plant-derived nanovesicles (PDNVs) have gained intense attention due to their distinguished biocompatibility, safety, cost-effectiveness, low immunogenicity, and easy accessibility [18, 19]. PDNVs entail a number of natural materials such as lipids, DNA, RNA, proteins, and other bioactive substances, which endow these nanovesicles with a variety of bioactivities including anti-inflammatory, anti‐oxidative, anti-cancer and immunoregulatory properties. These nanovesicles can be isolated or derived from a plethora of plants, such as ginger, ginseng, Catharanthus roseus and lemon which play important roles in cross-kingdom molecular exchange to modulate host immunity and exhibit therapeutic potential in a wide spectrum of diseases [20–24]. The key question to be addressed is whether PDNVs also have a role in modulating lung resident macrophages and promote the recovery of lung diseases, in particular ALI and ARDS, which have as yet no effective treatments.

In this study, we developed an approach to prepare the nano-size vesicles from *Artemisia annua*, a medicinal plant that has long been appreciated as an anti-malaria drug [25]. Emerging evidences have demonstrated that Artemisia and extracts possess multiple pharmacological properties for treating the inflammatory disorders, viral infections, autoimmunity, fibrosis, metabolic disorders and malignancy [26–29]. Of particular interest, recent studies reveal that Artemisia and the extracts display the therapeutic potential in treating severe respiratory diseases including COVID-19 [30–32], promoting us to further explore the material basis and mechanisms involved in this medicinal plant. Using three mice models of ALI, we herein demonstrate that Artemisia-derived nanovesicles (ADNVs) remarkably mitigated lung pathology and tissue damage caused by bacterial endotoxin, IAV and SARS-CoV-2 pseudovirus respectively. Importantly, we identify GABA as a key effector mediator that acted on AMs *via* GABA receptors to modulate the abundance and function of AMs. GABA-enclosing vesicles promoted mitochondrial genetic program, improved metabolic fitness, and reversed the inflammatory shift of AMs to resume lung homeostasis. The findings suggest a new and promising therapeutics for ALI by targeting mitochondrial metabolism of AMs, which may provide rationale for the development of *Artemisia*-based therapies to combat critical inflammatory lung diseases including COVID-19.

## Methods and materials

### Experimental animals

All animal experiments were approved by the Experimental Animal Ethics Committee of the Nanjing University of Chinese Medicine (approval number: 202312A007), and the principles and guidelines of the China Animal Protection Committee performed all procedures involved in animal experiments. Male C57BL/6 mice (6–8 weeks) were obtained from Anong Biotechnology Co., Ltd (Jiangsu, China).

### Cell lines and virus

Mouse alveolar macrophages (MH-S), Human embryonic kidney cells (293T) and Madin-Darby canine kidney (MDCK) cells were obtained from American Type Culture Collection (ATCC, USA). All cell lines were cultured in RPMI-1640 or DMEM medium containing 10% fetal bovine serum (FBS), 100 U/mL penicillin, and 100 µg/ mL streptomycin (all from Gibco, Carlsbad, CA, USA) in a semi-suspension and semi-adherent state. Influenza virus A/Puerto Rico/8/1934 (H1N1) (A/PR8) was expanded in MDCK cells, and the virus titer was determined by plaque assay. AdV-ACE2 adenovirus was purchased from Vita Bio (Shandong, China). The transfection reagent Lipo-2000 was co-transfected with plasmids Plenti-CMV-GFP-Puro (17,448, Addgene, USA), psPAX2(12,260, Addgene, USA), and SARS-CoV-2 spike (145,032, Addgene, USA) using 293T cells, and the supernatant was collected and concentrated to obtain SARS-CoV-2 pseudovirus.

### Isolation and purification of ADNVs

Take 100 g of fresh Artemisia annua branches and leaves, wash them three times with distilled water (ddH2O), and allow them to air-dry in a cool place. Combine the dried Artemisia annua branches and leaves with 300 mL of ddH2O in a juicer, extracting a liquid homogenate. Centrifuge the homogenate at 4000 × g for 1 h, collect the supernatant, and then centrifuge it again at 10,000 × g for 1 h. Transfer the resulting supernatant to an ultracentrifuge tube, centrifuge it at 100,000 × g for 2 h, and discard the supernatant. Dissolve the residue in sterile phosphate-buffered saline (PBS) solution, filter it through a 0.45 µM filter and collect it in a sterile centrifuge tube. Store the collected solution in a -80 °C refrigerator for future use.

### ADNVs labeling and ex *vivo* organ imaging

Take 200 mg of ADNVs, add 1 mM Dil staining solution, stain for 45 min at 37 °C under light-avoidance conditions (during which the tube was shaken intermittently to make the staining homogeneous), after staining, add sterile PBS, centrifuge at 100,000 × g for 2 h, discard the supernatant, and prepare it for use by washing twice with PBS. Dil-labeled ADNVs (25 mg/kg) were injected into mice *via* the tail vein. Imaging was performed by the IVIS Lumina imaging system (Xenogen Corporation, Hopkinton, MA, USA).

### Endotoxin-induced lung injury model

In vivo experiments, an acute lung injury model was established in C57BL/6 mice by indwelling needle airway perfusion injection of 1 mg/kg lipopolysaccharide 100 µL (O55:B5, Sigma, USA), and 4 h later, the mice were injected with 25 mg/kg of ADNVs through the tail vein, and 24 h later, the mice were killed for the subsequent experiments.

### Flow cytometry of BALF cells

Bronchoalveolar lavage fluid was collected and centrifuged at 1300 rpm for 5 min at 4 °C, and cells were resuspended in PBS for precipitation. The total number of cells in each mouse’s bronchoalveolar lavage fluid was counted under a microscope. The corresponding dose of streaming antibody alveolar macrophages (anti-CD45^+^, anti-CD11b^−^, anti-CD64^+^, anti-CD11c^+^, anti-SiglecF^+^), monocyte-derived macrophages (anti-CD45^+^, anti-F4/80^+^, anti-CD11b^+^), and neutrophils (anti-CD45^+^, anti-CD11b^+^, anti-Ly6G^+^) were successively added to each centrifuge tube. After thoroughly mixing the antibody with the cell suspension, the cells were incubated at 4 °C in the dark for 30 min and then detected by flow cytometry (BD Biosciences).

### Mito tracker green and red staining

According to the instructions of mitochondrial indicators Mito Tracker Red and Mito Tracker Green (1mM, all from Thermo, USA). The staining working solution was prepared using a ratio of 0.5 µM Mito Tracker-Red and 0.1 µM Mito-Tracker Green per milliliter of culture medium. Detection was performed using flow cytometry (BD Biosciences), and the data were saved and analyzed using Flow Jo software.

### mROS measurement

The cell precipitate was collected, added to 5 µM Mito-SOX staining solution (Thermo, USA) prepared, mixed thoroughly, incubated at 37 °C in the dark for 20 min. Cells were collected by centrifugation at 1500 rpm for 5 min and then washed three more times with PBS. The cells were resuspended by adding 200 µL PBS, and the precipitate was blown and mixed. The ROS levels were detected by flow cytometry (BD Biosciences) and analyzed by FlowJo software.

### Quantification of mtDNA levels

According to the manufacturer’s instructions, total DNA was isolated from cells using a FlexiGene DNA kit (Qiagen, Shanghai, China). Oligonucleotide Primer sequence probes targeting three different mitochondrial DNA (mtDNA) and one genomic DNA (gDNA) region were designed in Primer Bank. Quantitative real-time PCR was performed using Hieff qPCR SYBR Green Master Mix (Yeasen, Shanghai, China) to assess mtDNA content. Relative gene expression was analyzed using the ^△△^CT method using gDNA as an internal control to calculate the mtDNA copy number.

### ATP measurement

The cells were lysed sufficiently, centrifuged at 12,000 rpm and 4 °C for 5 min, and the supernatant was collected on ice and stored. ATP quantitative bioluminescence kit (Beyotime, Shanghai, China) was used for measurement on a multifunctional microplate reader (EnVision, USA).

### Quantitative realtime PCR (qRTPCR)

Cell sediments were collected, and RNA sediments were extracted using Trizol lysis (Thermo, USA). RNA samples were reverse transcribed according to the Reverse Transcription Kit (Yeasen, Shanghai, China). The reverse transcribed cDNA samples were diluted 5-10-fold with DEPC and stored at -20 °C. Next, real-time fluorescence quantitative PCR was performed using SYBR Green Master Mix (Yeasen, Shanghai, China) and determined on an ABI Prism 7500 Sequence Detection System (Applied Biosystems, USA). Quantitative expression levels were analyzed using the 2^−ΔΔCt^ method and normalized with β-actin. Primer sequences are shown in Table 1 of supplementary materials.

### Western blot assay

After lysing cells with RIPA (Beyotime, Shanghai, China) to obtain protein samples, protein concentration was measured using the BCA method. After electrophoresis, 5% skimmed milk was blocked for 2 h at room temperature, the primary antibody was incubated overnight at 4 °C, and the secondary antibody was incubated for 1 h at room temperature; anti-p38/p-p38, anti-ERK/p-ERK, anti-JNK/p-JNK, anti-p65/p-p65, anti-IKKα/p-IKKα, anti-IκBα/p-IκBα, anti-Sirt1, anti-β-Actin, anti-β-Catenin were obtained from Cell Signalling Technology (1:1000 dilution); anti-PGC1α, anti-COX15, anti-NDUFV2, anti-ATP5D, anti-ATP5H were obtained from Proteintech Biotechnology (1:1000 dilution); anti-TFAM was obtained from Absin Bioscience (1:1000 dilution); anti-p-GSK3β/GSK3β was purchased from Santa Cruz Biotechnology (1:1000 dilution); Peroxidase-conjugated secondary antibody (1:4000 dilution) was used, and ECL reagent (Beyotime, Shinghai, China) was added at a 1:1 ratio to develop the image on a digital imaging system (Bio-Rad, USA).

### Immunofluorescence

In cellular immunofluorescence staining, after 30 min of incubation of Dil-ADNVs with MH-S cells, Chlorpromazine hydrochloride (CPZ) at 10 µg/mL or Genistein at 200 µM (all from Aladdin, Shanghai, China) was added. After 4 h, samples were collected, and cells were fixed with 4% paraformaldehyde and washed thrice with PBS. The sections were blocked with 3% BSA for 1 h, washed 3 times with PBS, and treated with YF® 488-phalloidin overnight at 4 °C, washed 3 times with PBS, and incubated with Alexa Fluor 488 goat antibody for 1 h at room temperature, washed 3 times with PBS, and DAPI (1: 1000, Beyotime, Shanghai, China) were stained for 10 min, washed with PBS, and the cells were observed using a Leica TCS SP8 laser scanning confocal microscope after addition of an anti-fluorescence quenching agent. Primary rabbit antibody against F4/80 (1:200, Abcam, USA) was incubated overnight with frozen lung tissue sections at 4 °C, and Alexa Fluor 488 goat antibody was incubated for 1 h at room temperature. Nuclei were stained with DAPI (1:1000, Beyotime, Shanghai, China), washed three times with PBS, and imaged using a Leica TCS SP8 laser scanning confocal microscope (Leica Microsystems, Wetzlar, Germany).

### Viral infection

In vivo trials were conducted using male C57BL/6 mice aged 6–8 weeks. An influenza virus-infected mice model was used wherein each mouse received 50 µL of A/PR8 (1 × 10^4^/PFU) *via* airway drip. Four hours later, 25 mg/kg of ADNVs were administered *via* intravenous injection. Survival experiments demonstrated that the viral titer of A/PR8 was 1 × 10^8^/PFU. In the mouse model of pseudovirus infection, 25 µL/ mouse AdV-ACE2 adenovirus (1 × 10^10^ PFU/mL, WZ Biosciences, Shandong, China) was administered *via* airway drip. After 5 days, 50 µL/ mouse SARS-CoV-2 pseudovirus (1 × 10^6^ TCID50/mL) was instilled. ADNVs (25 mg/kg) was administered intravenously 2 days later.

### Macrophage depletion

After anesthesia, mice were perfused with 100 µL liposome chlorophosphoric acid solution (5 mg/mL, Yeasen, Shanghai, China) through the airway. The mice’s respiration, depth, and frequency were observed during the procedure. Mice in the non-macrophage-depleted group were also treated with PBS solution without liposomes. Subsequent experiments were performed 2 days after clearance.

### Adoptive transfer of macrophages

After macrophages were obtained from mouse bone marrow and cultured in vitro for 7 days, ADNVs (25 mg/kg) were incubated with macrophages (1×10^7^/mice). Two hours later, both types of macrophages treated with or without ADNVs were adoptively transferred into macrophage-depleted ALI model mice. Mice were euthanized and subjected to alveolar lavage and sampling.

### Influenza virus culture

MDCK cells were spread all over the six-well plate; after discarding the medium, sterile PBS was washed 3 times, 100 µL of BALF/virus infected supernatant to be tested was added, left at room temperature for 1 h, after discarding the supernatant, the supernatant was washed 3 times with PBS, and 2% agarose mixed with DMEM mono-culture 1:1 was added to the six-well plate, and then mixed well, and then placed at 37 °C for incubation, 3–5 days later, the number of empty spots was observed to calculate the virus titer.

### SARS-CoV-2 pseudovirus preparation

A 6-well plate was spread using 293T cells, and the transfection reagent Lipo-2000 (MCE, USA) was co-transfected with the plasmids pLenti-CMV-GFP-Puro (17,448, Addgene, USA), psPAX2(12,260, Addgene, USA), and SARS-CoV-2 spike (145,032, Addgene, USA). The supernatant was collected on days 2, 3, and 4 of the transfections, and PEG-8000 was added to 10% of the supernatant, spun at 4 °C overnight, and then centrifuged at 1000 g for 30 min. The precipitates were dissolved in 1% volume of a sample of the original supernatant in serum-free DMEM (+ F12) medium and stored at -80 °C for spare use.

### Statistical analysis

All statistical analyses were performed using GraphPad Prism software. Data are presented as mean ± SD of two or three independent experiments using two-tailed Student’s t-test, and one-way analysis of variance (ANOVA) followed by Bonferroni post hoc t-test. Survival curves were evaluated using Kaplan-Meier survival analysis and log-rank test. Values of *P* < 0.05 were considered significantly different.

## Results

### Preparation and characterization of ADNVs

**Fig. 1:**
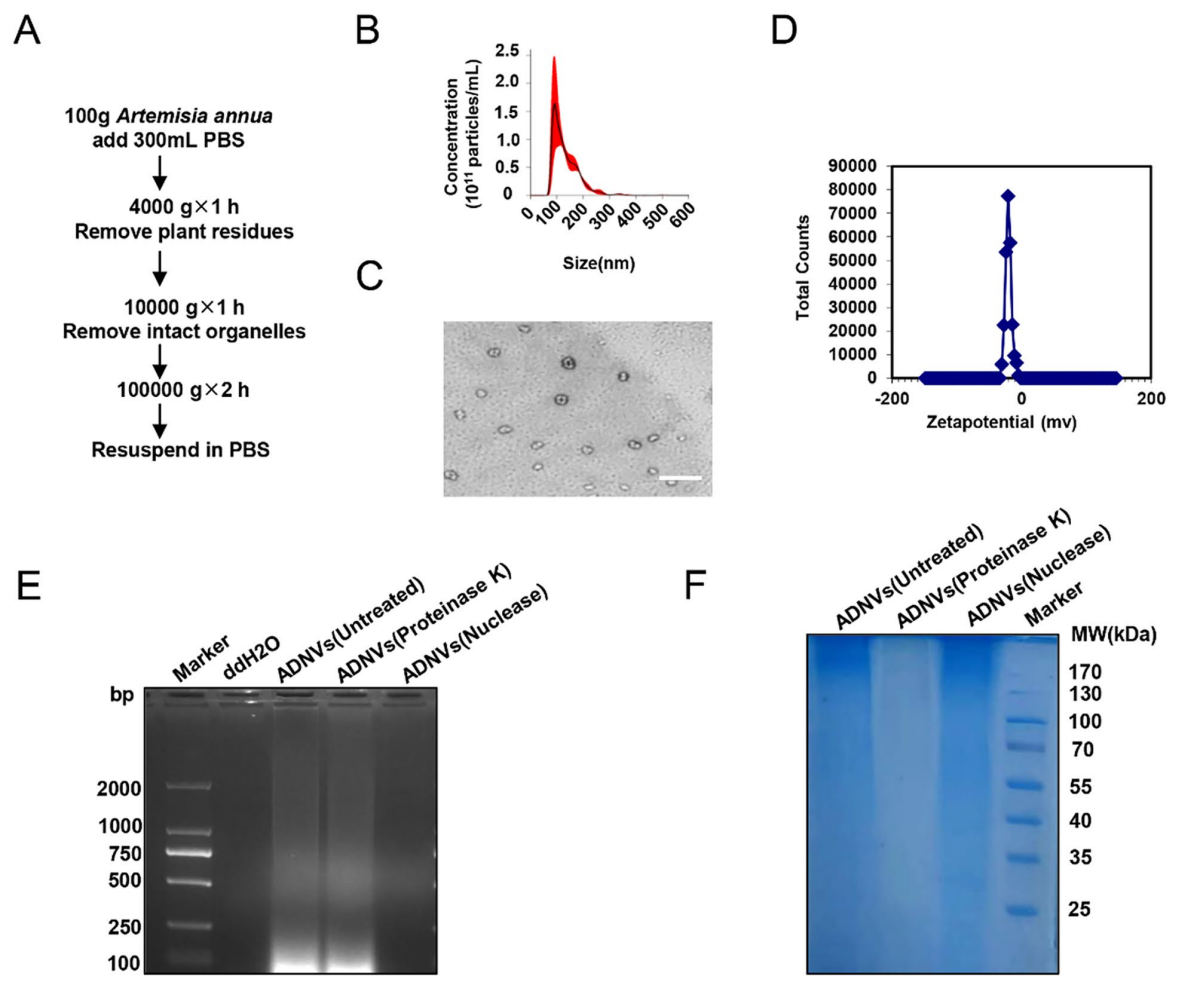
Isolation and characterization of ADNVs. **(A)** Flow chart of differential centrifugation procedure for ADNVs preparation. **(B)** Nanoparticle tracking analysis (NTA) determining the size and concentration of isolated ADNVs. **(C)** Transmission electron microscopy (TEM) of ADNVs. Scale bar, 200 nm. **(D)** Zetasizer measuring of the surface charges of ADNVs. **(E)** Agarose gel electrophoresis (1.2%) showing the DNA contents of ADNVs. Alternatively, ADNVs were pretreated with proteinase K (100 µg/mL), or nuclease (10 U/mL). DNA ladders as size markers. **(F)** The protein contents of ADNVs were detected by SDS-PAGE (10%) and Coomas Blue staining. Alternatively, ADNVs were pretreated with proteinase K (100 µg/mL), or nuclease (10 U/mL). Protein ladders as molecular weight (MW) markers. The results are one of three independent experiments. Shown are representative images

In this study, we fabricated the nanovesicles from *Artemisia annua L.* and evaluated its biological activity using mice models of acute lung injury (ALI). A modified protocol of sequential ultracentrifugation was applied to prepare Artemisia-derived nanovesicles (ADNVs) (Fig. 1A). Specifically, fresh *Artemisia annu*a was crushed and juiced, and then subjected to a low-speed centrifugation (4000 g, 1 h) to eliminate large plant residues, mucins, and fibers. The supernatants were then collected for medium-speed centrifugation (10,000 g, 1 h) to eliminate the intact organelles. The nanovesicles were finally obtained through ultra-high-speed centrifugation at 100,000 g for 2 h.

The biochemical and physical properties of ADNVs were subsequently characterized. Nanoparticle tracking analysis (NTA) revealed that ADNVs had an average diameter size of about 130 nm (Fig. 1B). The vesicles demonstrated round shapes and bilayer membranes, with a negative zeta potential value of − 22.5 mV (Fig. 1C, D). We additionally analyzed the compositions of ADNVs and revealed the presence of nucleic acids and protein-related substances, as revealed by agarose gel electrophoresis or SDS-PAGE electrophoresis respectively (Fig. 1E, F). Thus, we successfully isolated the nanovesicles from *Artemisia annua*, and demonstrated their unique properties and components.

### ADNVs are preferentially taken by lung macrophages to exert the immunoregulatory function

**Fig. 2:**
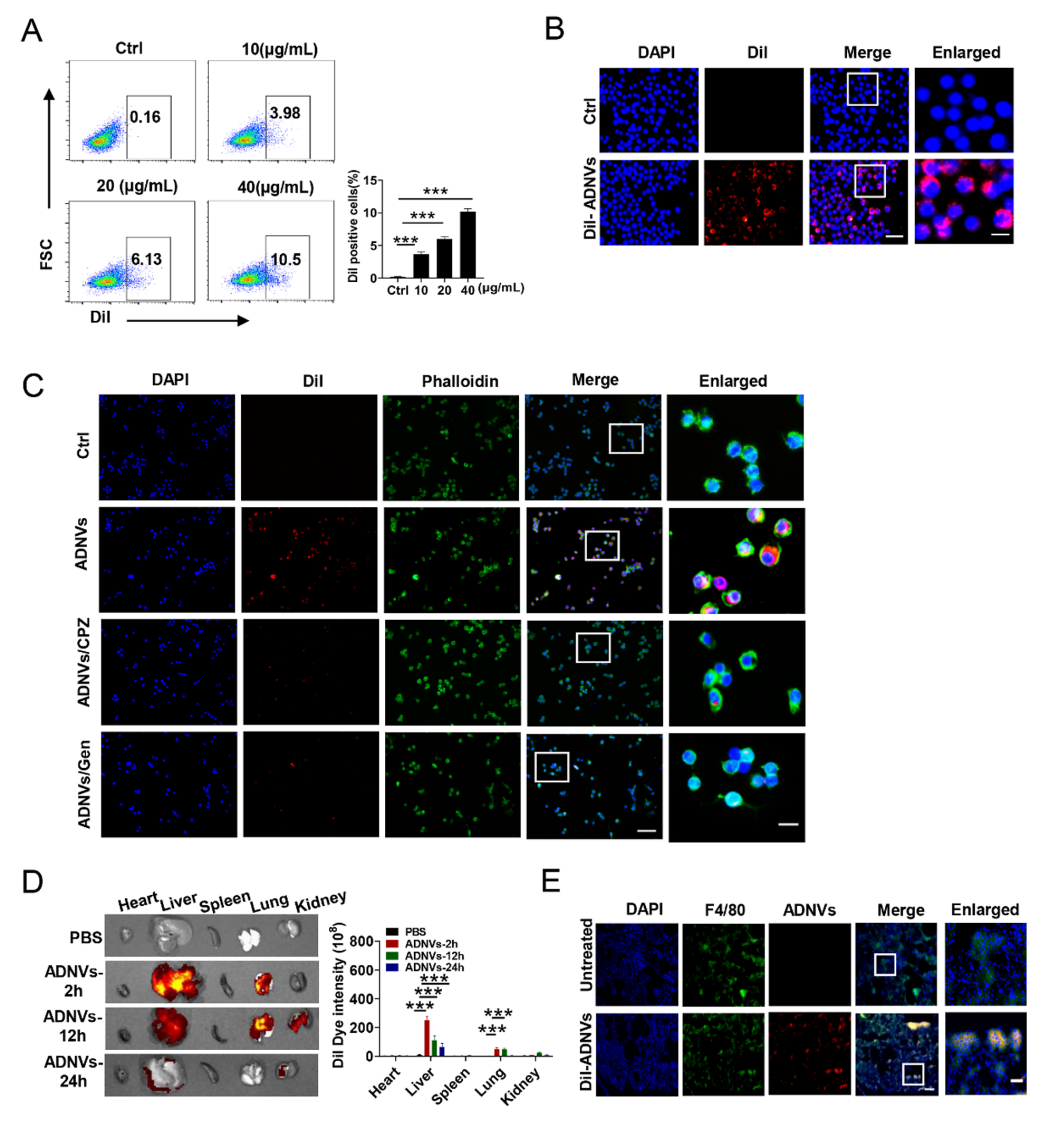
ADNVs are preferentially taken by macrophages. **(A)** Flow cytometry of the internalization of Dil-labeled ADNVs (10, 20 or 40 µg/mL) by MH-S cells. Cells were co-incubated with Dil-labeled ADNVs at the indicated doses for 4 h. **(B)** Representative confocal images showing the internalization of Dil-labeled ADNVs (20 µg/mL) by MH-S cells. DAPI, nuclear. Scale bar, 100/50 µm. **(C)** Representative confocal images showing the phagocytosis of ADNVs (20 µg/mL) by MH-S cells upon treatment of chlorpromazine (CPZ, 10 µg/mL) or genistein (Gen, 200 µM), or vehicle respectively. Phalloidin, F-actin; Scale bar, 200/50 µm. **(D)** Biodistribution of Dil-labeled ADNVs when injected into mice *via* the caudal vein by IVIS Lumina imaging system, and the quantitative analysis. **(E)** Representative confocal images showing the uptake of Dil-labeled by F4/80^+^ macrophages in lung tissue slices. Scale bar, 100/10 µm. The results are one of three independent experiments (A-C). Shown are representative images, and the data are expressed as the mean ± SD (A), *P < 0.05, **P < 0.01, ***P < 0.001

Macrophages are known as the first line of defense against invading pathogens, as well as a coordinator of the immune and inflammatory responses. We thus explored the potential role of ADNVs in regulating macrophages, particularly pulmonary macrophages that are critically involved in lung pathophysiology. Initially, ADNVs were labeled with Dil, a lipophilic membrane dye, and added to the culture of murine alveolar macrophage cell line, MH-S cells. Flow cytometry revealed a dose-dependent internalization of Dil-labeled ADNVs by MH-S cells, and confocal microscopy confirmed their uptake by macrophages (Fig. 2A, B). To understand how the nanoparticles were internalized by macrophages, we applied chlorpromazine (CPZ) or genistein (Gen), the specific blocker for clathrin- or caveolae-mediated endocytosis pathways, respectively [33]. The result showed that either of the inhibitors precluded the inclusion of ADNVs by macrophages was precluded by either of the inhibitors (Fig. 2C), implying the involvement of the two major mechanisms in this process.

Next, we examined the tissue distribution of ADNVs when intravenously injected into mice. The odyssey imaging analysis revealed that ADNVs rapidly trafficked to major tissues, livers and lungs particularly (Fig. 2D). Further examination of frozen sections of lung tissues demonstrated that Dil-labeled ADNVs were efficiently taken by F4/80^+^ macrophages (Fig. 2E). No evident histopathological changes were observed in the major organs including heart, liver, spleen, lung and kidney in ADNV-treated mice, and there was no significant difference in the levels of ALT, AST, and BUN in the serum compared to the PBS group, indicating the biosafety of the nanovesicles (Supplementary Fig. 1A, B). Together, the data indicated that ADNVs rapidly trafficked to lung tissues and were preferentially taken by macrophages.

The above findings promoted us to investigate how ADNVs impacted alveolar macrophages, the lung resident macrophages essential for tissue homeostasis. We utilized bacterial endotoxin, also lipopolysaccharide (LPS), to stimulate MH-S cells for evaluating ADNVs function. The data showed that ADNVs treatment markedly repressed the expression of pro-inflammatory factors including IL-1β, IL-6, TNF-α, and iNOS in LPS-stimulated macrophages (Supplementary Fig. 2A, G). Along with this, the activation of the nuclear factor kappa B (NF-κB) and mitogen-activated protein kinase (MAPK) signaling, the two major pathways driving the inflammatory response, were suppress by ADNVs administration (Supplementary Fig. 2B, C). Furthermore, LPS-induced M1 macrophages polarization (as defined by MHC-II^+^) was reversed to M2 type that was characterized by higher expression of CD206, arginase-1 (Arg-1), found in inflammatory zone-1 (Fizz-1), YM-1, transforming growth factor-β (TGF-β) and IL-10 (Supplementary Fig. 2D-F). Taken together, our data indicated that ADNVs were efficiently internalized by pulmonary macrophages, leading to the reprogramming of macrophages into the anti-inflammatory M2 type.

### GABA enclosed in the vesicles mediates the regulatory role of ADNVs

**Fig. 3:**
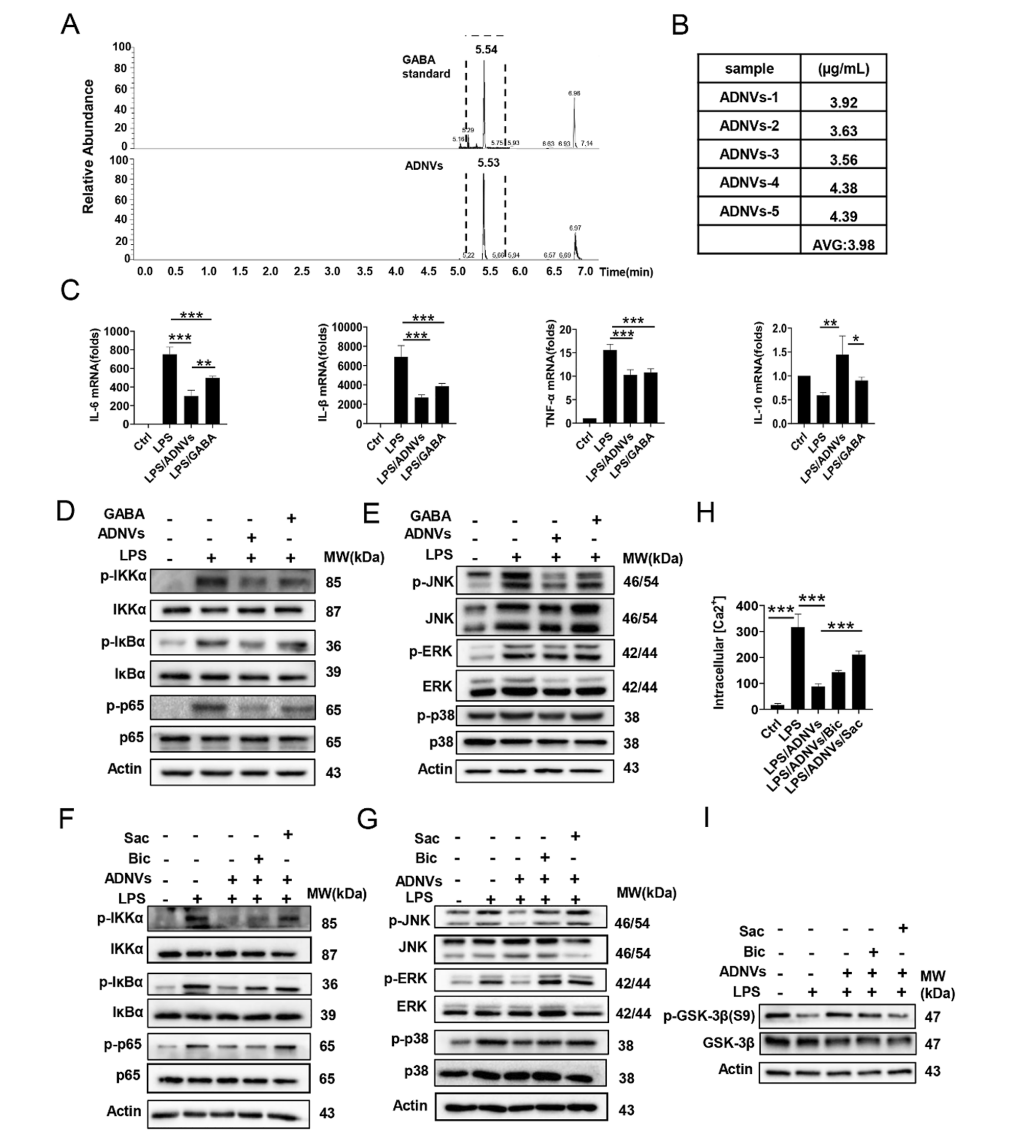
GABA-containing ADNVs suppress the inflammatory signaling. **(A, B)** GC-MS analysis of GABA in ADNVs according to the reference substance (A), and the concentrations were calculated. **(C)** qPCR analysis of the indicated cytokines in MH-S cells that were pre-incubated with ADNVs (GABA equal to 4 ng/mL) or GABA (100 nM), followed by LPS (100 ng/mL) stimulation for 4 h. **(D, E)** Western-blotting of the indicated signaling molecules in MH-S cells that were pre-incubated with ADNVs (GABA equal to 4 ng/mL) or GABA (100 nM) and then stimulated with LPS (100 ng/mL) for 4 h.**(F, G)**Western-blotting of the indicated signaling molecules in MH-S cells as described in D. Alternatively, bicuculline (Bic, 30 µM) or saclofe (Sac, 15 µM) were additionally added to the cellular culture. **(H)** The intracellular Ca^2+^ levels were measured as described in Methods. **(I)** Western-blotting of pGSK-3β/GSK-3β in MH-S cells as described in D. Bicuculline (Bic, 30 µM) or saclofe (Sac, 15 µM) were added in some circumstances. Molecular weights of the indicated molecules are depicted. The results are one of three independent experiments. Shown are representative images, and the data are expressed as the mean ± SD, *P < 0.05, **P < 0.01, ***P < 0.001

Next, we sought to identify the major components responsible for the immunoregulatory effect of ADNVs. Previous studies have reported that artemisinin acted through gamma-aminobutyric acid (GABA) pathway to modulate cellular differentiation and function, implying the involvement of GABA pathway in the action mode of Artemisia or related products [34]. GABA is a natural active ingredient released by plant and animal cells. Recent studies demonstrate that GABA exerts a range of bioactivities beyond the canonical neurotransmission role, playing a particular role in regulating the development and function of immune cells [35–37]. We thus hypothesized that Artemisia-derived vesicles might encase GABA to mediate the immunoregulatory effect. To test this, we utilized gas chromatography-mass spectrometry (GC-MS) to analyze the major components of ADNVs after sonication. Impressively, GABA was found to be encapsulated in the vesicles at a concentration range of 3.56–4.39 µg/mL, with the average concentration of 3.98 µg/mL (Fig. 3A, B). To test whether GABA in the vesicles was effective as it is, we then compared the effects of the enclosed GABA with the exogenously added GABA using LPS-stimulated MH-S cells. The results demonstrated that free GABA (100 nM, equivalent to 10.3 ng/mL) decreased the expression of IL-1β and TNF-α to the extent comparable to GABA-containing ADNVs (GABA, 4 ng/mL). Of interest, ADNVs treatment exhibited a more significant effect on the down-regulation of IL-6 and the up-regulation of anti-inflammatory IL-10 compared with exogenously added GABA (Fig. 3C). Similarly, GABA-containing ADNVs exerted more profound effects on the activation of NF-κB and MAPK pathways induced by LPS stimulation (Fig. 3D, E). At the same time, in the experiment of treating ALI model mice with the addition of exogenous GABA (supplementary Fig. 3A), exogenous GABA can alleviate the lung pathological injury of mice, reduce the total cell number and protein concentration in bronchoalveolar lavage fluid (BALF) (supplementary Fig. 3B-D), down-regulate the expression of pro-inflammatory factors IL-6, IL-1β and TNF-α, and up-regulate them levels of the anti-inflammatory factor IL-10 (supplementary Fig. 3E), while exogenous GABA treatment can increase the proportion of alveolar macrophages (AMs, CD45^+^CD64^+^CD11b^−^CD11c^+^SiglecF^+^) and reduce the number of monocyte-derived macrophages (MMs, CD45^+^F4/80^+^CD11b^+^) and neutrophils (CD45^+^Ly6G^+^CD11b^+^) (supplementary Fig. 3F-H).We thus proposed that the medicinal plant encased GABA and delivered it to macrophages for immune modulation.

To further confirm the functional relevance of the enclosed GABA, we subsequently evaluated the impact of the inhibition of GABA receptors on macrophages responses. It is currently known that two major receptors, GABA_A_ and GABA_B_ receptors, transduce the signaling initiated by GABA, with bicuculline (Bic) as GABA_A_R antagonist and saclofen (Sac) as GABA_B_R inhibitor [38]. Indeed, we showed that Sac administration largely abrogated ADNV-mediated suppression of inflammatory signaling with Bic treatment exerting the relatively moderate effect (Fig. 3F, G). The data thus indicated that ADNVs exerted the anti-inflammatory effects through engagement of GABA receptors, especially GABA_B_R. It has been reported that GABA could induce depolarization of the membrane potential of macrophages, leading to the inhibition of the inflammatory signaling [35]. In agreement with this, our data showed that LPS-induced intracellular Ca^2+^ flux was suppressed by ADNVs treatment and this effect was abrogated upon Sac treatment (Fig. 3H), further substantiating the involvement of GABA_B_ pathway in ADNVs effects. Notably, our data also demonstrated that treatment of ADNVs significantly elevated the inhibitory phosphorylation of GSK-3β (Ser9), indicative of the suppression of GSK-3β in LPS-stimulated macrophages (Fig. 3I). This effect was largely abrogated upon treatment of the GABA_B_R antagonist saclofen, reminiscent of the recent report about the down-regulation of GSK-3β by GABA_B_R signaling [36, 39]. Together, our data demonstrated that Artemisia-derived GABA was encapsulated in the vesicles to mediate the immunoregulatory effects *via* engagement of GABA receptors on macrophages.

### GABA-containing ADNVs improve mitochondrial integrity in stressed macrophages

**Fig. 4:**
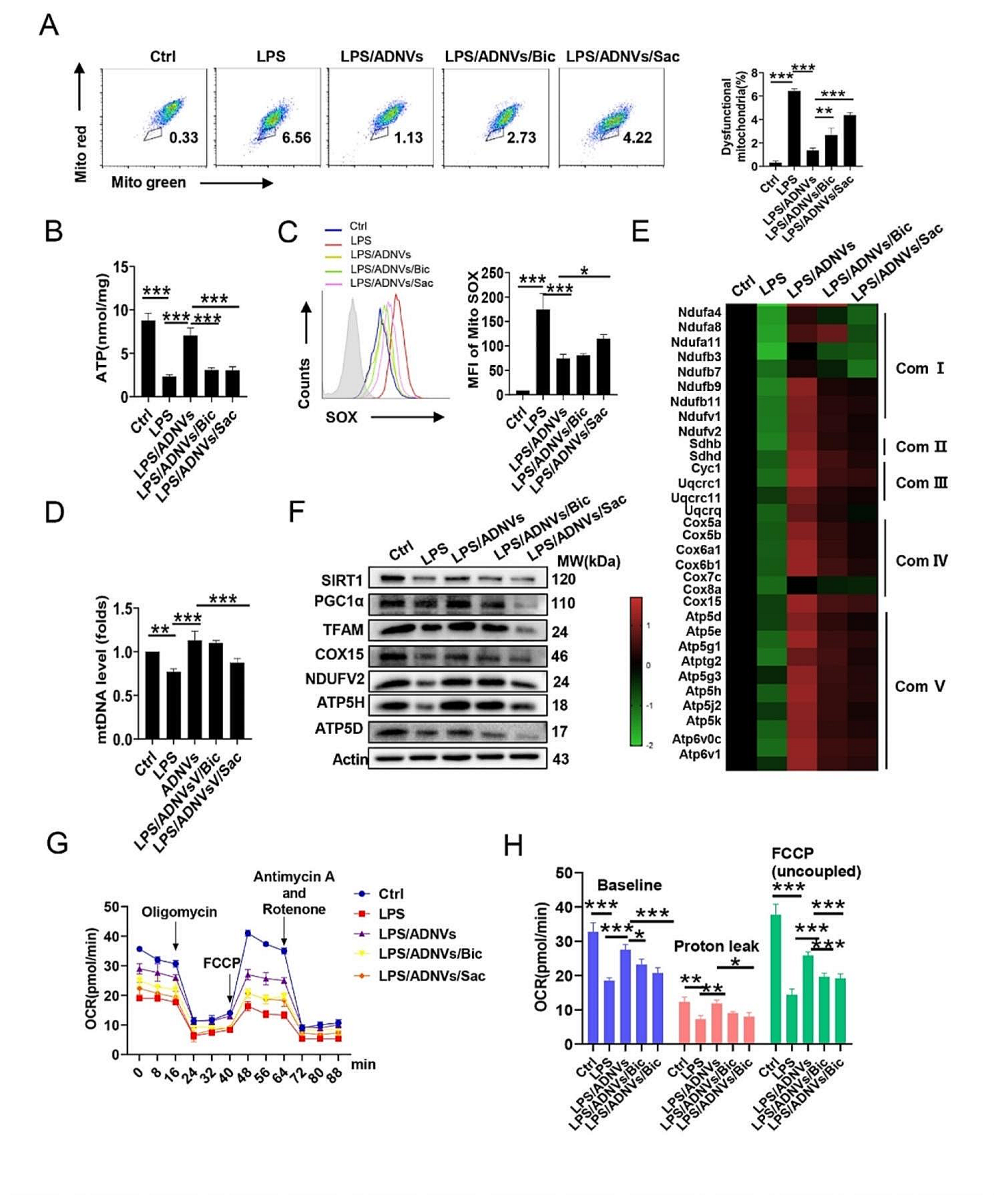
ADNVs corrected mitochondrial deficits in stressed macrophages. MH-S cells were pre-incubated with ADNVs (GABA equal to 4 ng/mL), followed by stimulation with LPS (100 ng/mL). In some instances, Bic (30 µM) or Sac (15 µM) were added. **(A)** Flow cytometry of the percentages of dysfunctional mitochondria (Mito-green^high^ Mito-Red^low^) in MH-S cells. **(B)** Mitochondrial ATP levels were measured. **(C)** Flow cytometry of mitochondrial ROS level. MFI, mean fluorescence intensity. **(D)** qPCR analysis of mitochondrial DNA levels. **(E)** The heat-map showing the expression of the representative genes of mitochondrial respiratory chain complexes I-V. **(F)** Western-blotting of the levels of mitochondria-related molecules. Molecular weights of the indicated molecules are depicted. **(G, H)** The oxygen consume rates (OCRs) were detected by Seahorse XFe96 Analyzer. The baseline respiratory capacity, proton leakage, and maximal respiratory capacity were calculated. The results are one of three independent experiments. Shown are representative images, and the data are expressed as the mean ± SD, *P < 0.05, **P < 0.01, ***P < 0.001

Emerging evidences have shown that mitochondrial integrity and function is essential for maintaining immune homeostasis and preventing exuberant inflammation and tissue damage. GABA has been documented to modulate multiple aspects of mitochondria, such as oxidative phosphorylation (OXPHOS), nucleoside salvage, TCA cycles and mitophagy [40, 41]. We then examined the mitochondrial function of AMs in LPS-stimulated mice with or without exogenous GABA treatment. Our data suggest that exogenous GABA can reduce the number of dysfunctional mitochondria (mito-Red^neg^-mito-Green^pos^), restoring levels of the mitochondrial respiratory complex (I-V) gene in AMs (supplementary Fig. 3I, J). We therefore set to explore the impact of GABA-containing ADNVs on mitochondrial activity. Remarkably, transmission electron microscopy (TEM) revealed that macrophages, following LPS stimulation, displayed disorganized mitochondria with swollen shapes and disrupted or absent cristae, whereas ADNVs treatment corrected this defection and improved mitochondria morphology featured with longer shape and organized cristae (supplementary Fig. 4A). The amounts of dysfunctional mitochondria (mito-Red^neg^ mito-Green^pos^)were markedly reduced by ADNVs treatment in LPS-stimulated macrophages. The effect however was abrogated upon blocking GABA receptors, particularly GABA_B_R, further supporting the implication of the GABAergic signaling in ADNVs action (supplementary Fig. 4B, Fig. 4A). In accordance, impaired ATP generation and exaggerated ROS production caused by LPS stimulation were corrected by ADNV administration depending on the intact GABA signaling (Fig. 4B, C). Addition of ADNVs also resumed the level of mitochondrial DNA (mtDNA) and mitochondrial respiratory complex (I-V) genes in LPS-stimulated macrophages (supplementary Fig. 4C-E, Fig. 4D, E). In accordance, the defective expression of the key molecules that control mtDNA replication and mitochondrial biogenesis, such as SIRT1, PGC-1α and TFAM, was elevated upon ADNVs administration (Fig. 4F). The effect however was compromised upon blockade of GABA receptors, particularly GABA_B_R. In parallel, LPS-induced decreased oxygen consumption rate (OCR), a hallmark of cellular mitochondrial function, was normalized by ADNVs treatment *via* GABAR engagement (supplementary Fig. 4F, Fig. 4G, H). Collectively, the results indicated that GABA-containing nanovesicles, primarily through GABA_B_R pathway, rectified defective mitochondrial activity, lessened oxidative stress, and thereby resumed macrophages homeostasis to prevent endotoxin-induced adverse effects.

### Delivery of ADNVs protects mice from acute lung injury *via* improving AMs fitness

**Fig. 5:**
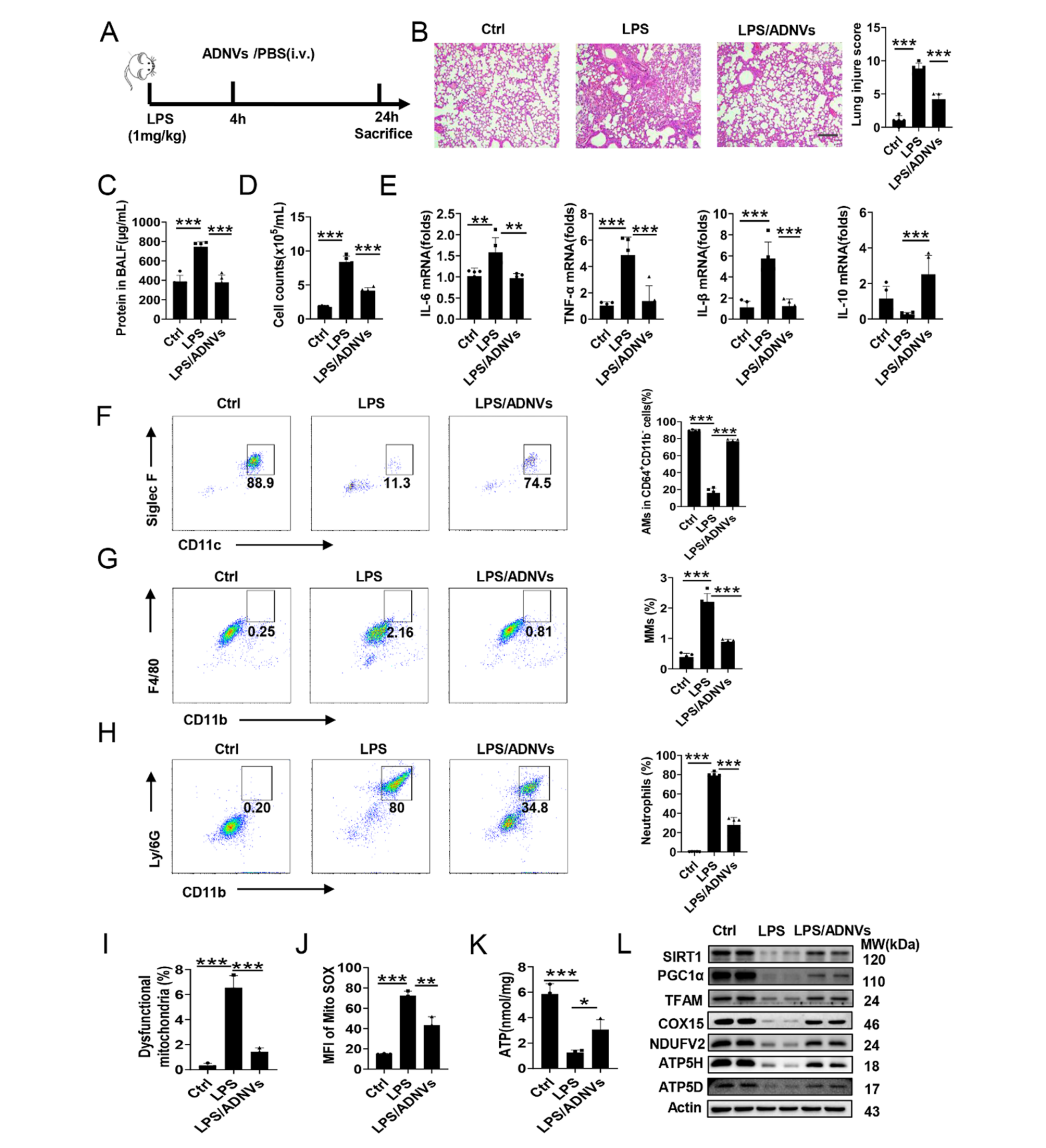
Administration of ADNVs alleviates endotoxin-induced lung injury in mice. **(A)** Simplified experimental scheme. C57BL/6 mice (*n* = 5) were intratracheally given LPS (1 mg/kg) for 4 h, and then treated with ADNVs (25 mg/kg, i.v.) or the vehicle (Ctrl). The mice were sacrificed 24 h later. **(B)** H&E staining of lung tissues and lung injury score. Scale bar, 200 μm. **(C)** Protein concentration, and **(D)** Total cell counts in BALF; **(E)** qPCR of the indicated cytokines in lungs; **(F-H)** Flow cytometry of the ratios of CD11c^+^SiglecF^+^ AMs (gated on CD45^+^CD11b^−^CD64^+^) (F), CD45^+^CD11b^+^F4/80^+^ MMs (G), and CD45^+^CD11b^+^Ly6G^+^ neutrophils (H) in BALF; **(I)** Flow cytometry of the percentages of dysfunctional mitochondria (Mito-Green^high^/Mito-Red^low^), and **(J)** the level of mitochondrial ROS. MFI, mean fluorescence intensity. **(K)** Measuring intracellular ATP levels. **(L)** Western-blotting of the representative mitochondrial genes. Molecular weights of the indicated molecules are depicted. Shown are representative images and the data are expressed as the mean ± SD, *P < 0.05, **P < 0.01, ***P < 0.001

Given the prominent roles for ADNVs in regulating AMs activity and their enrichment in lung tissues when administrated intravenously (Fig. 2D, E), we speculated that the vesicles might have the regulatory role in pulmonary immunopathology. To test it, we established a mice model of acute lung injury (ALI) by intratracheal instillation of endotoxin, and ADNVs were administrated to evaluate their effects (Fig. 5A). As expected, histological examination revealed that endotoxin-challenged mice exhibited thickening alveolar walls, disorganized pulmonary structure and increased cellular infiltration. Treatment of ADNVs however significantly alleviated the pulmonary immunopathology, characterized by improved lung structural integrity and lessened the inflammatory cells infiltration (Fig. 5B). Consistently, counts of total cell and proteins leaked in bronchoalveolar lavage fluid (BALF), indicative of increased endothelial permeability, were reduced upon ADNVs administration in endotoxin-challenged mice (Fig. 5C, D). Also, ADNVs administration caused a profound decrease in the expression of pro-inflammatory cytokines IL-6, TNF-α, and IL-1β, but elevated the level of anti-inflammatory factor IL-10 (Fig. 5E). Together, the results demonstrated that ADNVs treatment significantly alleviated lung inflammation and improved lung pathology in endotoxin-challenged mice.

As is known, lung macrophages are heterogeneous with different subsets having distinct phenotypes and functions [6]. Specifically, lung resident alveolar macrophages (AMs) are essential for pathogens clearing and tissue repairing, whereas monocyte-derived macrophages (MMs) contributed significantly to lung immunopathology [5–7, 42]. Our data demonstrated that intratracheal instillation of endotoxin caused a remarkable decrease in the proportion of CD45^+^CD64^+^CD11b^−^CD11c^+^SiglecF^+^AMs, and increased the percentages of CD45^+^F4/80^+^CD11b^+^ MMs and CD45^+^Ly6G^+^CD11b^+^neutrophils, the two major proinflammatory cell subsets underpinning acute lung injury. Strikingly, ADNVs administration reversed this trend and greatly increased ratio of AMs and reduced the infiltration of MMs and neutrophils in lungs (Fig. 5F-H). The data thus indicated that ADNVs exerted the regulatory effect on alveolar macrophages, and potentially other innate immune cells.

Given the importance of functional mitochondria in maintaining macrophage fitness and robustness, we then examined the mitochondrial status of AMs in LPS-challenged mice with or without ADNVs treatment. Remarkably, the data showed that, compared with those from vehicle-treated endotoxic mice, AMs from ADNV-treated mice displayed significantly reduced amounts of functional mitochondria with reduced mROS release and boosted ATP generation (Fig. 5I-K). In parallel, the defective expression of mitochondria-supporting factors SIRT1, PGC-1α and TFAM, as well as that of mitochondrial respiratory chain complex genes such as COX15, NDUFV2, ATP5H and ATP5D, was largely resumed upon ADNVs treatment (Fig. 5L). The results were in line with the in vitro findings and supported the mitochondria-protecting effect of ADNVs (Fig. 4A-C, F). Together, our data demonstrated that ADNVs treatment improved mitochondria integrity and AMs fitness, thereby ameliorating lung immunopathology and protecting mice from endotoxin-induced ALI.

### Alveolar macrophages are required for the protective effects of ADNVs

**Fig. 6:**
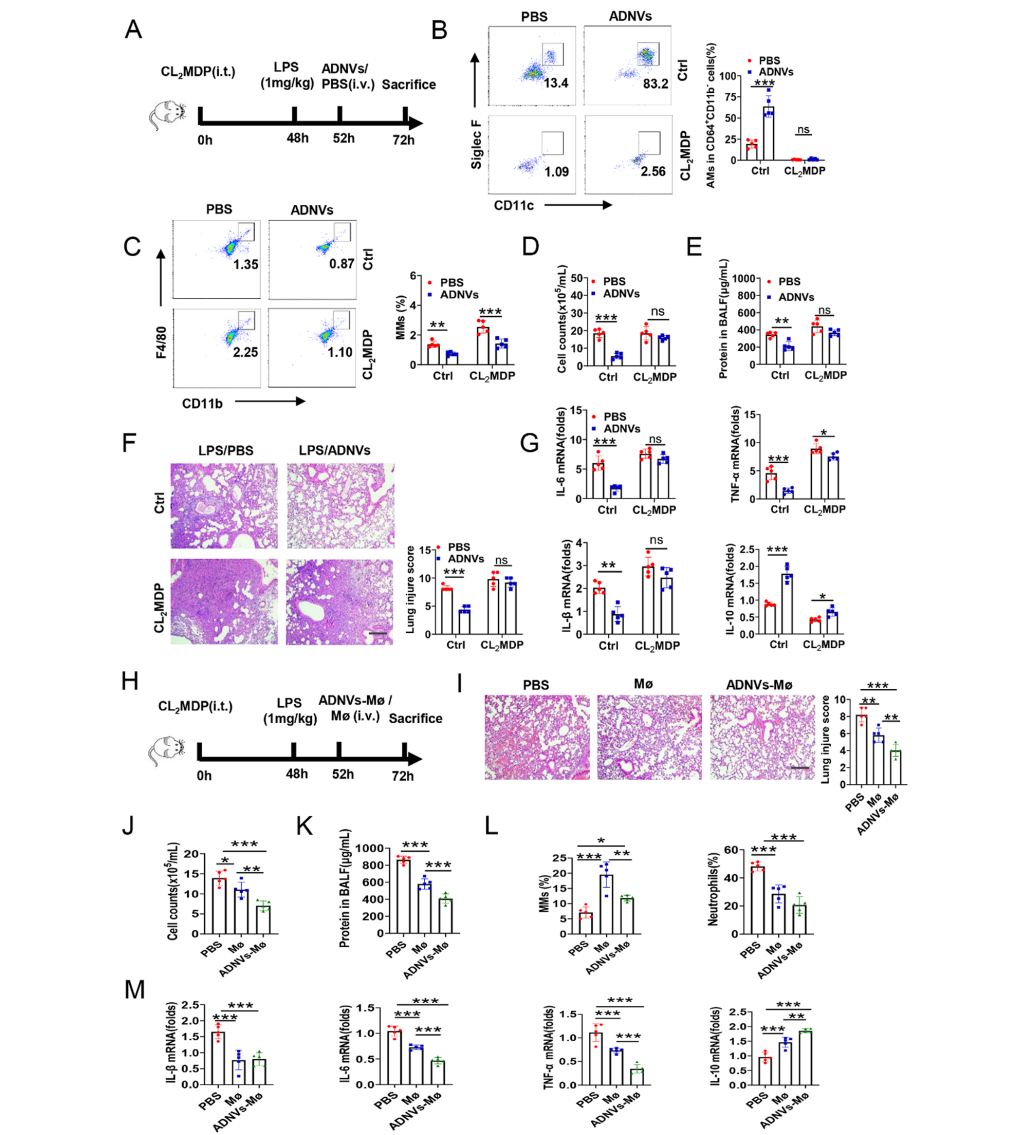
Alveolar macrophages are required for the protective effects of ADNVs. **(A-G)** Simplified scheme of AMs depletion experiment. C57BL/6 mice (*n* = 5) were intratracheally instilled with 100 µL CL_2_MDP (5 mg/mL) for 2 days. The mice were then treated with LPS (1 mg/kg, i.t.), followed by ADNVs administration (25 mg/kg, i.v.). The animals were sacrificed 24 h and subjected to functional analysis **(A)**; **(B)** Flow cytometry of CD11c^+^SiglecF^+^ AMs gated at CD45^+^CD11b^−^ CD64^+^; **(C)** Flow cytometry analysis of MMs (CD45^+^CD11b^+^ F4/80^+^); **(D)** Total cell counts, and **(E)** Protein concentration in BALF; **(F)** H&E staining of lung tissues and lung injury score. Scale bar, 200 μm; **(G)** qPCR of the indicated cytokines in lung tissues. **(H-M)** The simplified scheme of macrophage adoptive transfer experiment. Murine bone marrow-derived macrophages (BMDMs) were prepared and pre-conditioned with or without ADNVs for 2 h. The mice (*n* = 5) were subjected to LPS (1 mg/kg) challenge, 4 h later, the cells were then adoptively transferred to mice that were pre-depleted pulmonary macrophages. **(I)** H&E staining of lung tissues and lung injury score (Scale bar, 200 μm); **(J)** Total cell counts, **(K)** Protein concentration, and **(L)** the ratios of macrophages and neutrophils in BALF; **(M)** qPCR of the indicated cytokines in lungs tissues. Shown are representative images and the data are expressed as the mean ± SD, *P < 0.05, **P < 0.01, ***P < 0.001

Alveolar macrophages are a resident population comprising over 90% of total cells in healthy lungs, playing a pivotal role in orchestrating the immune and inflammatory response to maintain tissue homeostasis [5, 6]. To confirm the direct effect of ADNVs on AMs, we then applied chlorophosphate liposomes (CL_2_MDP), a well-established macrophage removing agent [43], to deplete AMs and assess the effect of ADNVs (Fig. 6A). As expected, pre-conditioning of CL_2_MDP almost completely cleared AMs (CD45^+^ CD64^+^CD11b^−^CD11c^+^SiglecF^+^), but enhanced the infiltration of proinflammatory macrophages (CD11b^+^F4/80^+^) (Fig. 6B, C). In parallel, total cells infiltrated in lung tissues, the protein leaked in BALF, as well as the expression of proinflammatory cytokines were elevated, while the level of IL-10 was increased upon AM removal (Fig. 6D-E, G). These mice also exhibited exaggerated lung pathologies despite the administration of ADNVs (Fig. 6F). Thus, our data indicated that depletion of AMs compromised the effect of ADNVs in alleviating lung inflammation and injury caused by endotoxin, supporting the requirement of AMs for fulfilling ADNVs effects.

Furthermore, to give direct evidence for macrophage-targeting effects of ADNVs, we additionally conducted adoptive transferring experiments. For this, bone marrow-derived macrophages (BMDMs) were prepared, reconditioned with ADNVs, after 4 h of endotoxin stimulation, the mice were adoptively transferred to pre-depleted lung macrophage mice. (Fig. 6H). The results demonstrated that, compared with vector-treated macrophages, ADNV-primed macrophages markedly improved lung tissue integrity, reduced total cell counts, BALF protein content and the inflammatory cell infiltration, and also suppressed the expression of inflammatory cytokines but elevated the level of the anti-inflammatory cytokine IL-10 (Fig. 6I-M). Together, our data supported that alveolar macrophages were indispensable and essential for ADNVs to achieve their immunoregulatory and lung-protecting effects.

### ADNVs administration protects mice from influenza pneumonia and confers survival benefits

**Fig. 7:**
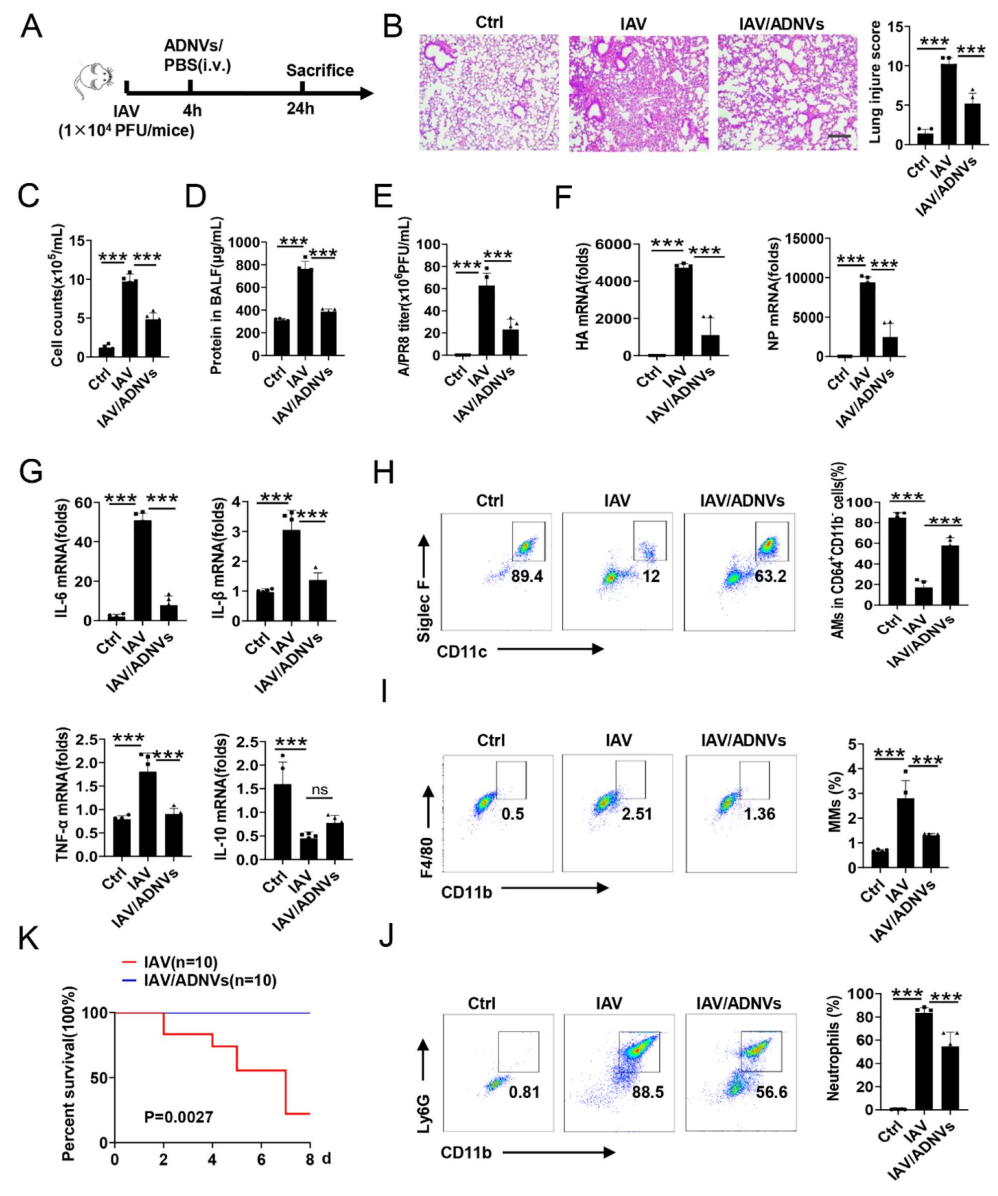
ADNVs mitigate influenza pneumonia and increase the survival rate. **(A-J)** Schematic diagram of the experiment. C57BL/6 mice (*n* = 5) were intratracheally instilled with influenza A virus strain A/PR8 (10^4^ PFU/mice), followed by ADNVs administration (25 mg/kg, i.v.). 24 h later, the mice were sacrificed for alveolar lavage and sampling. **(B)** H&E staining of lung tissues and lung injury score. Scale bar, 200 μm; **(C)** Total cell counts, **(D)** protein concentrations, and **(E)** Viral loads were assessed in BALF; qPCR assay of the levels of **(F)** HA and NP and **(G)** the cytokines as indicated in lungs; Flow cytometry analysis of the ratios of **(H)** CD11c^+^SiglecF^+^AMs gated in CD45^+^CD11b^−^CD64^+^, **(I)** CD45^+^CD11b^+^F4/80^+^ macrophages, and **(J)** CD45^+^CD11b^+^Ly6G^+^ neutrophils. **(K)** Kaplan Meier survival analyses of the mice challenged with the lethal dose of A/PR8 strain (10^8^ PFU/mice) prior to ADNVs treatment or not (*n* = 10). Log-rank test *P* < 0.01. Shown are representative images and the data are expressed as the mean ± SD, *P < 0.05, **P < 0.01, ***P < 0.001

Influenza virus poses a serious global health threat with causing half a million deaths every year, and the deadly cases are frequently associated with deregulated AMs and unchecked inflammation [44]. We thus wondered whether ADNVs could confer a protection against influenza pneumonia. To this end, mice were infected with influenza PR8 strain, followed by ADNVs administration (Fig. 7A). The results showed that ADNVs treatment significantly improved lung pathology, reduced cell infiltration, and lessened BALF protein leakage in IAV-infected mice (Fig. 7B-D). Concurrently, viral loads were greatly reduced upon ADNV treatment, correlating with decreased levels of viral core proteins, hemagglutinin (HA) and nuclearprotein (NP) (Fig. 7E, F). Administration of ADNVs also reduced the expression of proinflammatory cytokines, accompanied with resumption of AMs abundance and decreased infiltration of MMs and neutrophils (Fig. 7G-J). Thus, the data showed that ADNVs treatment provided the protection for mice against influenza pneumonia by resuming AMs homeostasis and tissue integrity. In support, application of ADNVs significantly elevated the survival rate of mice that were challenged with a lethal dose of IAV (Fig. 7K).

### ADNVs treatment improves lung pathology caused by SARS-CoV-2 pseudovirus

**Fig. 8:**
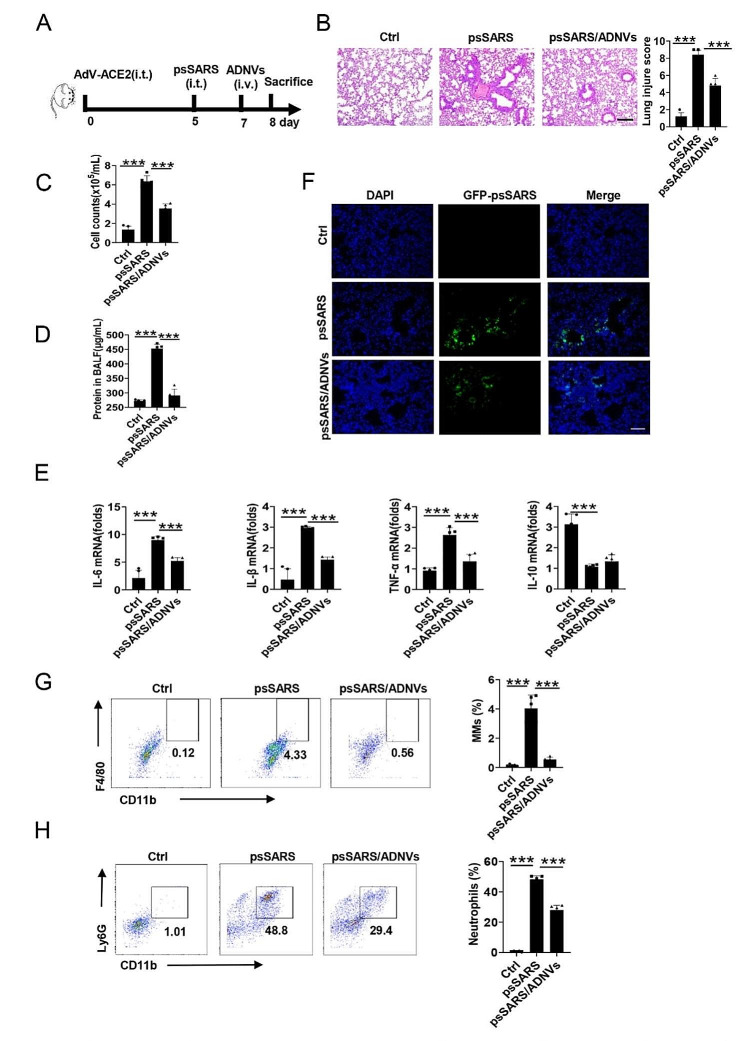
ADNVs confer the protection for mice from SARS-CoV-2 pseudovirus infection. **(A)** Simple flowchart of the experiment. Mice were firstly infected with hACE2-expressing adenovirus (AdV-ACE2, 2.5×10^8^ PFU/mice). 5 days later, the mice were injected with pre-made SARS-CoV-2 pseudovirus (psSARS) (5 × 10^4^ PFU/mice), followed by administration of ADNVs (25 mg/kg) or PBS (Ctrl), and 24 h later, mice were sacrificed for alveolar lavage and functional analysis (*n* = 5). **(B)** H&E staining of lung tissues and lung injury score. Scale bar, 200 μm. **(C)** Total cell counts, and **(D)** Protein concentrations in BALF. **(E)** qPCR assay of the indicated cytokines in lungs. **(F)** Confocal image showing the psSARS loads in frozen lung sections. DAPI, nucleus; GFP, psSARS. Scale bar,100 μm. Flow cytometry analysis of the ratios of **(G)** CD45^+^CD11b^+^F4/80^+^ macrophages, and **(H)** CD45^+^CD11b^+^Ly6G^+^ neutrophils in BALF. The representative images are shown and the data are expressed as the mean ± SD, *P < 0.05, **P < 0.01, ***P < 0.001

Accumulating evidences have linked heightened innate immunity and impaired mitochondrial function with severe COVID-19 and poor prognosis [45, 46]. To evaluate whether the benefits of ADNVs would extend to SARS-CoV-2 infection, we established a mouse model of SARS-CoV-2-pesudovirus (psSARS) infection, a reliable and feasible approach widely used to study SARS-CoV-2 pathology or screen the relevant treatments [47, 48]. Since C57BL/6 mice did not express human ACE2, a cognate receptor for SARS-CoV-2 viruses, we firstly constructed a replication-defective hACE2-expressing adenovirus and infected mice with AdV-hACE2. 5 days later, mice were challenged with vesicular stomatitis virus (VSV)-based SARS-CoV-2 pseudovirus (psSARS) that were prepared as described in [48]. The mice were then divided into two groups for receiving ADNVs or the vehicle respectively (Fig. 8A). Remarkably, the histological examination revealed that intratracheal instillation of psSARS caused moderate but significant lung inflammation and injury, along with an elevation in cells infiltration, protein leakage and proinflammatory cytokines production (Fig. 8B-E). The data was reminiscent of the observation about the pathogenic altercation caused by SARS-CoV-2 infection in hACE2 transgenic mice [49]. ADNVs treatment also reduced the viral burden, as demonstrated by weakened florescent intensities due to GFP-tagged psSARS (Fig. 8F). Concurrently, the ratios of proinflammatory cell subsets, MMs and neutrophils, were significantly repressed in ADNV-treated mice (Fig. 8G, H). Thus, our data demonstrated that ADNVs treatment abated the cytokine storm-like pathology and improved lung tissue integrity, conferring a profound protection for mice against psSRAR-CoV-2 infection.

## Discussion

Dysfunctional mitochondria and distorted alveolar macrophages are critically involved in the pathogenesis of ALI and ARDS, which provides the rationale for developing mitochondria and AM-targeting treatments for severe respiratory diseases [12, 13, 15, 16]. In the present study, we isolated nanovesicles from the medicinal plant, Artemisia, and demonstrated their alleviating roles in alleviating lung inflammation and injury mainly through the regulation of AMs abundance and function. Importantly, we identified GABA in ADNVs as a major effector factor that functioned to rectify mitochondrial genetic defects, oxidative stress, and energetic insufficiency *via* the engagement of GABA receptors, and thereby restore AMs fitness for preventing extensive lung damages. Given the prominent importance of AMs in regulating lung pathophysiology and the currently lack of corresponding specific therapeutics, this finding may open a new avenue for the development of treatments for the clinically relevant lung diseases, such as endotoxin-induced lung injury, influenza pneumonia, and COVID-19.

Owing to their distinct anatomical location and functional importance, AMs are important for host defensive responses, which are however vulnerable to the infectious and inflammatory insults. In agreement with the previous reports [8, 15, 50], our data demonstrated that the amounts and phenotypes of AMs were profoundly affected by endotoxin and virus challenges. In fact, recent data have revealed that exacerbated pathogenesis of COVID-19 was causatively related with dramatic diminishment of AMs [51]. The transition of these lung tissue-resident macrophages from the anti-inflammatory toward pro-inflammatory phenotype has been reported to contribute significantly to the immunopathology of COVID-19 [52, 53]. Accordingly, the resumption of functional AMs was proposed as a driving force for COVID-19 recovery, justifying the development of the therapeutics aimed to correct dysfunctional macrophages to alleviate viral pneumonia. As a proof of concept, our data show that ADNVs treatment substantially inhibited the inflammatory signaling, resumed the abundance of AMs, promoted macrophages shift from MHC-II^hi^ M1 to CD206^hi^Arg-1^hi^IL-10^hi^ M2 type [9], and thereby greatly improved lung pathologies in several mice models of ALI. Although the M1/M2 dichotomy seems too simplified to exactly describe the spectrum of lung macrophages that appeared during the response to invading pathogens or other challenges, emerging evidences have linked the overriding of M2 to M1 macrophages with lessened pulmonary immunopathology [54]. Interestingly, besides the pro-inflammatory property, M1-polarized macrophages have recently been reported to promote virus replication and maturation because of their lower endosomal pH that promotes membrane fusion and viral RNA release. By contrast, M2-type AMs can potentially impede viral invasion because they generally deliver virus to acid lysosomes for degradation and eradication [55]. These findings may explain our discovery of decreased viral loads in ADNV-treated mice by correlating the phenotypic shift of AMs towards the M2 type as evidenced in this study.

To confirm the functional relevance of AMs to ADNVs action mode, we additionally conducted macrophage depletion and adaptive transfer experiments. Notably, we found that ablation of AMs caused an increased influx of monocyte-derived macrophages (MMs) and neutrophils in ADNV-treated endotoxic mice. These results are in line with the recent findings illustrating that AMs play a central role in maintaining lung immune homeostasis, while their ablation would induce enhanced infiltration of peripheral MMs and even the replacement of resident macrophages by recruited macrophages [56]. Even though, at this stage we cannot completely exclude the possibility that ADNVs may have direct effects on other innate immune cells such as MMs or neutrophils, a question meriting further investigations in future studies.

The sustainability of AMs populations and their function are determined by multiple factors. Well-organized mitochondria are of particular importance for AMs homeostasis especially in stressed lungs, because the macrophages need more bioenergetics and metabolic intermediates to support their self-renewal and synthesis of immunoregulatory factors to restrain the infections and resume tissue integrity [57]. Rectifying mitochondrial deficits in diseased lungs is therefore crucial for restoring AMs function and resolving the inflammation. Despite accumulated data supporting the therapeutic values of PDNVs, only few current studies have reported their effects on mitochondria and AMs. We herein provide the enticing evidences that ADNVs potentially improve mitochondrial genetic program, enhance OXPHOS levels and ATP generation, and resume redox balance in the functionally compromised AMs in challenged mice. Such reparative effect is causatively related with the modulation of mitochondrial genetic program and biogenesis, the major events that are frequently disrupted by the infectious and injurious insults [10, 14, 58–60]. Of interest, we noted that defective expression of TFAM (a nuclear-encoded transcription factor controlling mtDNA replication and stability), and PGC-1α (a transcriptional co-activator of TFAM) was greatly improved by ADNVs treatment in LPS-stimulated macrophages. In line with our current report, studies have shown that TFAM deficiency causes mitochondria damage and mtDNA depletion, which would in turn lead to cellular energetic insufficiency, oxidative stress, and mtDNA stress-mediated inflammatory responses underpinning the development of severe pulmonary diseases such as influenza pneumonia and COVID-19 [61–63]. Given the central importance of TFAM-driven mitochondrial genetic program in regulating cellular metabolism and function, it is tempted to speculate that modulation of TFAM and related pathways contributes, at least partially, to the mitochondria-supporting effects of ADNVs. Future studies might be needed to further explore detail molecular mechanisms responsive for ADNVs-mediated mitochondrial responses in AMs.

In this study, we not only elucidate the action mode and targeting cells of ADNVs, but also identify GABA as the enclosed effector factor that is delivered by the vesicles to mediate the interkingdom crosstalk and immunomodulatory function. Though GABA is generally recognized as a neurotransmitter, the molecule exhibits multiple bioactivities including the regulation of immune cells such as CD4^+^ and CD8^+^ T cells, tumor-associated macrophages, etc [35, 36, 41]. . Our present study further expands the GABA-targeted cell type by showing its regulatory role in AMs through the modulation of mitochondrial function and inflammatory signaling. On the other hand, ADNVs are double-membrane bound structure, making GABA more stable and resistant to enzyme digestion or acid microenvironments when administrated in vivo, more efficiently to be delivered to distal organs and internalized by innate immune cells, and more safer to avoid potential off-target effects [35, 64]. As proof-of-concept evidence, our data demonstrated that, our data demonstrated that, compared with free GABA (100 nM, equal to10.3 ng/mL), GABA enclosed in ADNVs (equal to 4 ng/mL) exhibited greater effects on the regulation of LPS-induced inflammatory signaling and proinflammatory cytokines production. In addition, enclosed GABA has a more profound effect in reducing the production of pro-inflammatory IL-6 and increasing the release of anti-inflammatory IL-10. Indeed, the appropriate expression of IL-10 has been linked with the survival profits of patients with sepsis, and its ablation led to exaggerated pulmonary inflammation and deadly sepsis [65]. It is therefore reasonable to speculate that increased level of IL-10 may underlie the lung-protecting effects of ADNVs. Moreover, our data illustrated that the induction of IL-10 correlated well with the inhibition of GSK3β, in agreement with the recent finding that GSK3β exerted an inhibitory effect on IL-10 expression *via* interacting with c-Maf and Blimp-1 [66]. On the contrary, GABA-enclosed ADNVs displayed to profoundly suppress the expression of IL-6, a canonical proinflammatory cytokine that has been depicted as a signature of ARDS and a biomarker of cytokine storm characterizing severe COVID-19 pathologies [67, 68]. Given the paramount importance of these cytokines in regulating lung immunopathology, intense efforts are nowadays made to develop IL-6 or IL-10-based treatments. Therefore, our discovery of GABA-enclosed nanovesicles may represent an optimal therapeutics approach for pulmonary inflammatory diseases, given their excellent biocompatibility, biosafety, and efficacy [17–19].

Another important finding in our present study is the identification and confirmation of the dominant role of GABA in mediating the immunoregulatory role of ADNVs. Although some other substances encased in ADNVs may possibly have a biological role, we herein provides the enticing evidences to support the functional importance of GABA in the vesicles. In particular, our data indicate that inhibition of GABA receptors largely eliminates the inhibitory effect of ADNV-mediated inflammatory signals and pro-inflammatory cytokines, and this inhibition also abolishes its role in improving mitochondrial integrity and activity. Furthermore, we show that inhibition of GABA_B_ receptor (GABA_B_R) yielded more significant effects than blockade of GABA_A_ receptor (GABA_A_R), indicating that GABA_B_R pathway essentially mediated ADNVs downstream signaling. This argument is further supported by the observed GSK-3β activation, a proinflammatory factor acting downstream and repressed by GABA_B_R pathway [36, 69], was down-regulated upon ADNVs treatment. Although the causes for such differential GABA receptors activation capacity of ADNVs are currently unknown, studies have demonstrated that the two GABA receptors exhibited differential sensitivity to their ligands with GABA_B_R response to a much lower dose of ligand than GABA_A_R [70]. The findings may provide a plausible explanation for the preferential activation of GABA_B_R by ADNVs, as the working concentration of enclosed GABA in this study is as low as 4 ng/mL. Thus, the data suggest the high sensitivity and selectivity of ADNVs, which endows the vesicles with the therapeutic efficacy as well as the biosafety to avoid inadvertent activation of GABA pathway that may cause neurological, immunologic and other systems disorders [35, 36, 40].

In conclusion, *Artemisia annua* is a widely used medicinal plant with a great range of biological activities including anti-malarial, anti-cancer, antioxidant, anti-inflammatory and anti-microbe’s properties [26–29, 31, 71, 72]. Broader application of Artemisia however is hindered by several potential problems, such as the still elusive mechanism, the multiplex constituents, and the potential side effects. The present study demonstrates the potential roles for artemisia-derived nanovesicles in mitigating lung pathology and promoting disease recovery *via* regulating mitochondrial integrity and AM fitness. Our findings provide insight into the pathogenesis of ALI and ARDS, and open a new avenue for the development of effective treatments for critical respiratory diseases such as COVID-19.

### Electronic supplementary material

Below is the link to the electronic supplementary material.

Supplementary Material 1

## Data Availability

The data that support the findings of this study are available from the corresponding authors upon reasonable request.
