# Supplementary material for: Modulation of alveolar macrophage and mitochondrial fitness by medicinal plant-derived nanovesicles to mitigate acute lung injury and viral pneumonia
Source: J Nanobiotechnology. 2024 Apr 18;22:190. doi: 10.1186/s12951-024-02473-w (PMC11025283; doi:10.1186/s12951-024-02473-w)
Supplement: Supplementary file 1 — Supplementary Material 1 [file 12951_2024_2473_MOESM1_ESM.docx]

**Supplementary Information**

**Modulation of alveolar macrophage and mitochondrial fitness by medicinal plant-derived nanovesicles to mitigate acute lung injury and viral pneumonia**

Lusha Ye^1,2^, Yanan Gao^2^, Simon Wing Fai Mok^3^, Wucan Liao^2^, Yazhou Wang^2^, Changjiang Chen^2^, Lijun Yang^1^, Junfeng Zhang^2^, Liyun Shi^1,2, #^

**
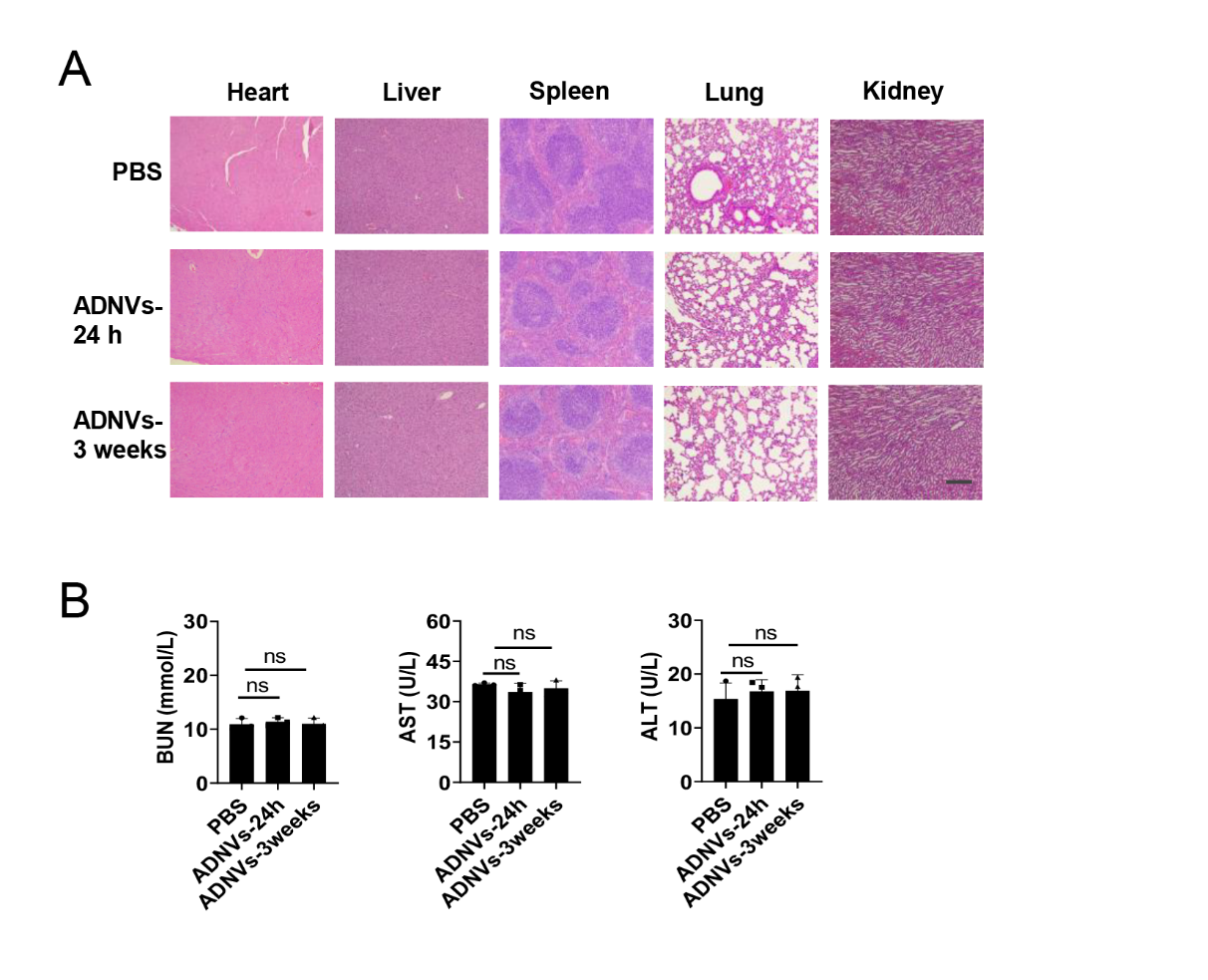
**

**Supplementary Figure 1. ADNVs exhibit no significant tissue toxicity.** C57BL/6 mice (n=3) were given LPS (1 mg/kg, i.t.), followed by administration of ADNVs (25 mg/kg) or PBS via the tail vein 4 h later. (A) The major organelles including heart, liver, spleen, lung and kidney were harvested 24 h or 3 weeks post inoculation, and subjected to H&E staining and microscopy examination. Scale bar, 200 μm. (B) Levels of AST, ALT, and BUN in mice serum. Shown are the representative images, and the data are expressed as the mean ± SD; **P* < 0.05, ***P* < 0.01, ****P* < 0.001.

**
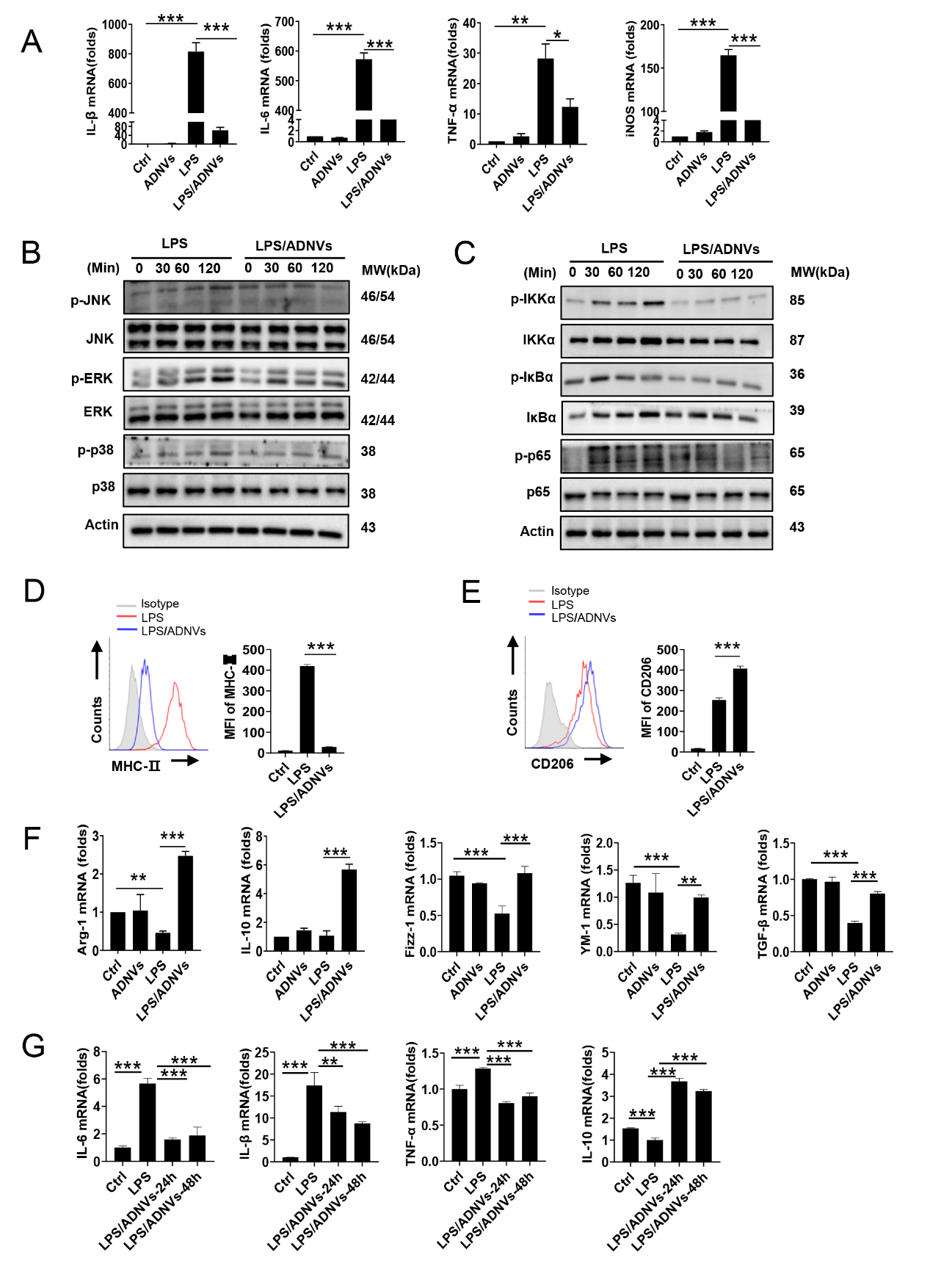
**

**Supplementary Figure 2. ADNVs exert the anti-inflammatory effects in LPS-stimulated macrophages.** MH-S cells were pre-incubated with ADNVs (GABA equal to 4 ng/mL) or the vehicle (Ctrl) for 30 min, and stimulated with LPS (100 ng/mL) for 4 h. Cells were collected for subsequent analysis. **(A)** qPCR assay of the indicated cytokines. **(B, C)** Western-blotting of the signaling proteins related with NF-κB and MAPK pathways. **(D, E)** Flow cytometry of the levels of MHC-II and CD206. MFI, mean fluorescence intensity. **(F)** qPCR of Arg-1, Fizz-1, YM-1, TGF-β and IL-10 levels. The results are from three independent experiments. **(G)** MH-S cells were pre-incubated with ADNVs (GABA equal to 4 ng/mL) or the vehicle (Ctrl) for 30 min, and stimulated with LPS (100 ng/mL) for 24 h or 48h. qPCR of IL-6, IL-1β, TNF-α and IL-10 levels. Shown are the representative images and the data are expressed as the mean ± SD, **P* < 0.05, ***P* < 0.01, ****P* < 0.001.


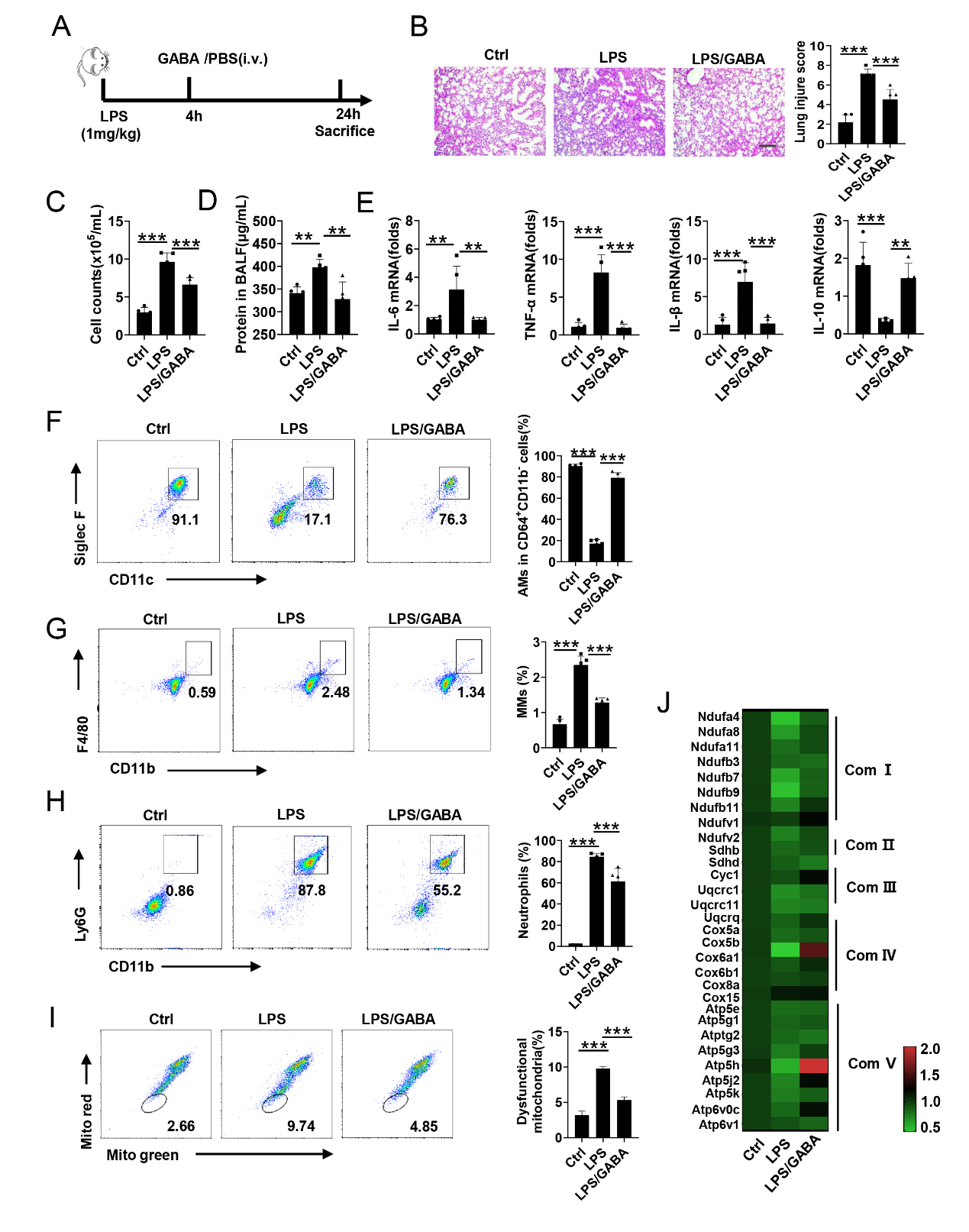


**Supplementary Figure 3. Administration of** **exogenous GABA alleviates endotoxin-induced lung injury in mice. (A)** Simplified experimental scheme. C57BL/6 mice (n=5) were intratracheally given LPS (1 mg/kg) for 4 h, and then treated with exogenous GABA (100nM, i.v.) or the vehicle (Ctrl). The mice were sacrificed 24 h later. **(B)** H&E staining of lung tissues and lung injury score. Scale bar, 200 μm. **(C)** Protein concentration, and **(D)** Total cell counts in BALF; **(E)** qPCR of the indicated cytokines in lungs; **(F-H)** Flow cytometry of the ratios of CD11c^+^SiglecF^+^ AMs (gated on CD45^+^CD11b^-^CD64^+^) (F), CD45^+^CD11b^+^F4/80^+^ MMs (G), and CD45^+^CD11b^+^Ly6G^+^ neutrophils (H) in BALF; **(I)** Flow cytometry of the percentages of dysfunctional mitochondria (Mito-Green^high^/Mito-Red^low^), and **(J)** Heatmap showing the levels of mitochondrial respiratory chain complex I-V genes. Shown are representative images and the data are expressed as the mean ± SD, **P* < 0.05, ***P* < 0.01, ****P* < 0.001.

**
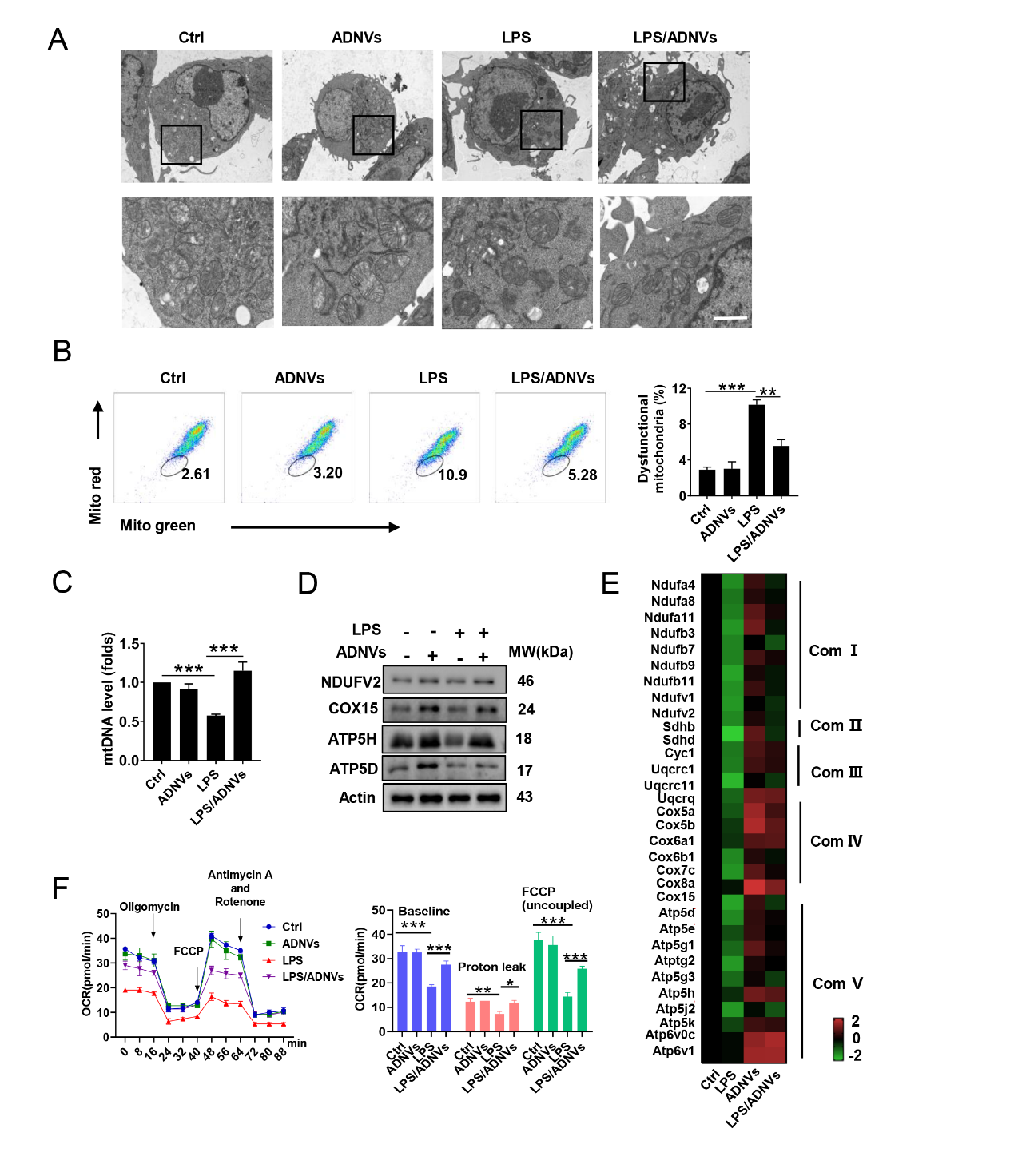
**

**Supplementary Figure 4. ADNVs treatment improves the mitochondrial function in LPS-stimulated macrophages**. MH-S cells were pre-incubated with ADNVs (GABA equal to 4 ng/mL) or the vehicle (Ctrl) for 30 min, followed by stimulation with LPS (100 ng/mL) for 4 h. (**A)** Representative transmission electron micrographs (TEM) images showing mitochondrial amount, morphology and cristae. Scale bar, 1μm. **(B)** Representative flow cytometry charts illustrating percentage of dysfunctional mitochondria (Mito-Green^high^Mito-Red^low^). **(C)** qPCR assay of mitochondrial DNA. **(D)** Western-blotting of mitochondrial-associated proteins. The molecular weights of the indicated molecules are depicted. **(E)** Heatmap showing the levels of mitochondrial respiratory chain complex I-V genes. **(F)** The oxygen consume rates (OCRs) were examined by Seahorse XFe96 Analyzer. The baseline respiratory capacity, proton leakage, and maximal respiratory capacity were calculated. The results are from three independent experiments. Shown are the representative images, and the data are expressed as the mean ± SD; **P* < 0.05, ***P* < 0.01, ****P* < 0.001.

**Supplementary Methods**

**Characterization of ADNVs**

Extracted of fresh ADNVs, sterile PBS uniform injection diluted, Nanosight NS300 instrument (Malvern, UK) set the following parameters: the camera 6, gain 1, 12, detection threshold exposure 5. Three videos were captured at a time, and the average of the measurements made through the three videos was the final detection result. ADNVs were stained with 2% uranyl acetate saturated alcohol solution in the dark, and their morphology was imaged in a JEM-1010 electron microscope (JEOL, Tokyo, Japan).

**Zeta potential measurement**

ADNV samples were prepared and sonicated for 2 min to maintain the samples in a uniform distribution without precipitation or aggregation, placed in a measuring instrument Zetasizer Nano ZS90 (Malvern，UK), and zeta potential values were calculated from the measured potential change data.

**Nucleic acid electrophoresis**

A 1.2% agarose gel was configured, and samples treated with or without nuclease (10 U/mL) were loaded into each well using GL DNA Marker 2000 (AG, Hunan, China). Images were displayed by a digital imaging system (Bio-Rad, USA) after a 45 min electrophoresis time.

**Coomassie Blue Staining**

Fresh ADNVs were extracted, and the protein concentration in ADNVs was detected by the BCA Protein Concentration Assay Kit (Beyotime, Shanghai, China). ADNVs samples treated with or without proteinase K (100 μg/mL) were added to the electrophoresis hole, and the voltage of the concentrated gel was 90 V, and 120 V was used for separating the gel. The electrophoresis was terminated until the bromophenol blue was still about 1 cm away from the bottom of the glass plate. Gel was placed into glassware, and Caulmers Brilliant stain solution (Beyotime, Shanghai, China) was added and placed on a shaker at room temperature for 90 min. Caulmers Brilliant staining solution was recovered, and the gel was washed with water. Add Caulmers Brilliant for decolorization night and place on a decolorization shaker for decolorization overnight. The decolorization solution was removed, and the gel was rinsed with water before the target bands were photographed.

**Measurement of intracellular Ca^2+^ concentrations**

MH-S cells were preincubated with ADNVs (GABA equal to 4 ng/mL) for 30 min, and then 100 ng/mL LPS was added. Bicuculline (30 μM, MCE, USA) or saclofen (15 μM, MCE, USA) were added together. The cell precipitate was collected 4 h later for subsequent processing. MH-S cells were washed with sterile PBS, and then 2 μmol/L of Fura-2/AM (MCE, USA) was added to the cell suspension and incubated at 37 °C for 45 min. The suspension was centrifuged at 1200×g for 10 min at 4 °C, washed twice with PBS, and left for 20 min. Fluorescence values (F) were measured using a fluorescence spectrophotometer at an excitation wavelength of 340 nm, an emission wavelength of 500 nm, and a slit width of 10nm. TritonX-100 (Amrisco, USA) at a final concentration of 0.2% was then added to the suspension to measure Fmax. 5 mmol/L EGTA (Solarbio, Beijing, China) was added to the cell suspension containing TritonX-100 to measure Fmin. [Ca^2+^] i was calculated according to the following formula (Kd:224 nmol/L): [Ca^2+^] i (nmol/L) = Kd🞨((F−Fmin)/(Fmax−F)).

**Hematoxylin-eosin staining**

Lung tissue was fixed with 4% paraformaldehyde, dehydrated, and cut into 3-5 μM sections after dewaxing and hydration, stained with hematoxylin for 2-3 min, rinsed with tap water for 5 min, and then stained with eosin for 10 s, immersed in tap water and rinsed for 5 min, with a gradient of alcohols from low to high, and immersed in 70%, 80%, 90%, 95%, 100% Ⅰ, and 100% Ⅱ alcohols in sequence for 1 min, and then successively placed into the stained xylene Ⅰ, and Ⅱ, and immersed in each of them for 10 min. The slices were sealed by neutral tree resin, and the changes in the morphology of the lung tissues were observed under the microscope (Leica, Wetzlar, Germany). The four indexes of alveolar and interstitial inflammation, edema, hemorrhage and thickening of alveolar septum were evaluated by semi-quantitative scoring system: 0 score, no damage in visual field; 1 score, lesion range < 25%; 2 scores, the lesion range ≥25%~< 50%; 3 scores, the lesion range ≥50%~< 75%; 4 marks, ≥75%~ full field of vision. Each score was added to the total lung injury score, and the average of the two researchers independently scored the lung injury as the final score.

**Bronchoalveolar lavage**

After the mice were euthanized, the limbs were fixed, and the needle head of the indwelling needle was fixed in the trachea at the incision of the trachea using a "0" suture. The mice's bronchoalveolar lavage fluid (BALF) was collected by perfusion of 1 mL PBS through the trachea cannula. The supernatant was aspirated by centrifugation at 1300 rpm for 5 min at 4 °C and stored in a refrigerator at -80 °C until use. The total protein in BALF was determined according to the BCA protein assay kit (Beyotime, Shanghai, China).

**Isolation and culture of primary mouse alveolar macrophages**

Collect groups of mice alveolar lavage fluid, absorbing supernatant, after centrifugal -80 ℃ refrigerator spare, with 10% FBS and 100 U/mL penicillin, and 100 μg/ mL streptomycin of DMEM medium heavy precipitation suspension cells, the cell suspension inoculation to six orifice, The cells were incubated at 37 ℃ in a constant temperature incubator with volume fraction of 5% CO_2_ for 2 hours. After 2 hours, the culture supernatant was discarded, gently washed twice with sterile PBS solution. After centrifugation, the primary mouse alveolar macrophages were obtained. This study was to prepare for the subsequent experiments of Mito tracker green and red staining, ATP level, mtDNA copy number, reactive oxygen species function and mitochondrial complex Western blot in alveolar macrophages.

**Extracellular flux analysis**

The Seahorse XFe96 Extracellular Flux Analyzer (Seahorse Bioscience, USA) characterized the metabolic state of the cells by detecting changes in oxygen consumption rate (OCR) and extracellular acidification rate (ECAR) in real time under basal conditions and after the addition of different drugs to the culture medium. OCR was used to study mitochondrial oxidative phosphorylation function. This was determined by measuring basal respiration in the normal state (basal respiration), followed by the addition of oligomycin to inhibit ATP synthetase, followed by the addition of the uncoupling agent FCCP, and finally by the addition of the electron transfer inhibitor antimycin A. The concentrations of oligomycin (1 μM), FCCP (0.5 μM), antimycin A (1 μM), and rotenone (100 μM) were prepared according to the manufacturer's instructions.

**Transmission electron microscopy (TEM)**

Cells were first fixed with an electron microscope fixative at 4 °C for 12 h, preembedded in AGAR, and fixed with 1% osmic acid at room temperature in the dark for 2 h. The samples were successively dehydrated in 30%-50%-70%-80%-95%-100%-100% alcohol for 20 min each time and 100% acetone twice for 15 min each time. After infiltration and embedding, the plate was placed in the oven at 37 ℃ overnight, and the plate was placed in the oven at 60 ℃ for polymerization for 48h. The resin block was removed for use. The resin blocks were sliced on an ultra-thin microtome (LEICA EM UC7, Wetzlar, Germany) and scoped out with a 150-square Chinese membrane copper mesh. The copper mesh was stained with 2% uranyl acetate saturated alcohol solution in the dark for 8min, washed three times with 70% alcohol, and three times with ultra-pure water. The copper mesh was stained with 2.6% lead citrate solution in the dark for 8min, washed three times with ultra-pure water, and the filter paper was slightly blotted dry. The copper mesh sections were dried overnight in the copper mesh box at room temperature. Observations were made under a transmission electron microscope JEM-1230 (JEOL, Tokyo, Japan), and images were collected and analyzed.

**Isolation and culture of mouse bone marrow-derived macrophages**

Bone marrow cells were extracted from 8-week-old C57BL/6 mice, precipitated by repeated blowing with DMEM complete medium through a 200-mesh sieve; the filtrate was collected and centrifuged, the supernatant was discarded, and resuspended and spread in a petri dish by the addition of DMEM complete medium containing M-CSF (25 ng/mL), and the cells were collected on day 7.

**GC-MS**

ADNVs were extracted and sonicated for 8 min on ice, and then evaporated in a centrifugal concentrator (45 ℃, 15kpa), 500 μL of methanol (Merk, USA) was added to the evaporated samples, vortexed for 3 min, centrifuged at 18,000 rpm for 10 min at 4 ℃, 400 μL of supernatant was aspirated, and 10 μL of methanol solution containing 300 μg/mL of 1,2-13C myristic acid (Sigma, USA) was added, vortexed for 5 min, and then placed in a centrifugal concentrator to evaporate (45 ℃, 15kpa). 30 μL of methoxyamine pyridine solution (10 mg/mL, Sigma, USA) was added to the evaporated sample. The sample was vortexed for 5 min, and then the sample was oscillated at 300 rpm in a thermostatic oscillator at 30 ℃ for 1.5 h. Add 30 μL of BSTFA (Sigma, USA), vortex for 1 min, oscillate at 300 rpm in a thermostatic oscillator, oscillate at 37 °C for 0.5 h, centrifuge at 18000 rpm, and centrifuge at 4 °C for 10 min, and then aspirate the supernatant of 40 μL into the injection vial for sample analysis. The analytical instrument used in this experiment was a Trace 1310-TSQ8000 Evo triple quadrupole gas chromatograph (Thermo Fisher, USA). The analytical conditions were set as a TG-5MS capillary column (0.25 mm × 30 m × 0.25 μm), with the gradient heating procedure: 0~1 min, 100 ℃; 1~9.8 min, 100 ~320 ℃; 9.8~14.8 min, 320 ℃, shunt ratio of 20:1, injection volume of 1 μL. The EI ion source, ionization energy of 70 eV, ion transfer line temperature of 250 ℃, ion source temperature of 280 ℃, mass spectrometry was full-scanning mode, the scanning range of 50-500; the carrier gas was high-purity helium (purity > 99.999%); the carrier gas flow rate was 1.2 mL The flow rate of the carrier gas was 1.2 mL/min. GABA: tR:6.54min, daughter and parent ion: 174.1-73, CE: 14eV; ISTD: tR:6.97min, daughter and parent ion: 119.1-75, CE: 10eV.

**Abbreviations**

| ALI | Acute lung injury |
| --- | --- |
| AMs | Alveolar macrophages |
| IAV | Influenza A virus |
| ADNVs | Artemisia-derived nanovesicles |
| GAGA | Gamma-aminobutyric acid |
| ARDS | Acute respiratory distress syndrome |
| MMs | Monocyte-derived macrophages |
| ATP | Adenosine triphosphate |
| TCA | Tricarboxylic acid |
| PDNVs | Plant-derived nanovesicles |
| LPS | Lipopolysaccharides |
| MH-S | Mouse alveolar macrophages |
| FBS | Fetal bovine serum |
| MDCK | Madin-Darby canine kidney cells |
| 293T | Human embryonic kidney cells |
| ddH2O | Distilled water |
| PBS | Phosphate-buffered saline |
| BALF | Bronchoalveolar lavage fluid |
| mtDNA | Mitochondrial DNA |
| CPZ | Chlorpromazine hydrochloride |
| OCR | Oxygen consumption rate |
| TEM | Transmission electron microscopy |
| A/PR8 | Influenza virus A/Puerto Rico/8/1934 |
| DMEM | Dulbecco’s Modifed Eagle’s Medium |
| GC-MS | Gas chromatography-mass spectrometry |
| NTA | Nanoparticle tracking analysis |
| CPZ | Chlorpromazine |
| Gen | Genistein |
| NF-κB | Nuclear factor kappa B |
| MAPK | Mitogen-activated protein kinase |
| Arg-1  Fizz-1  TGF-β | Arginase-1  Found in inflammatory zone-1  Transforming growth factor-β |
| Bic | Bicuculline |
| Sac | Saclofen |
| OXPHOS | Oxidative phosphorylation |
| TEM | Transmission electron microscopy |
| CL_2_MDP | Chlorophosphate liposomes |
| HA | Hemagglutinin |
| NP | Nuclearprotein |

**Table 1 Primer sequences for quantitative RT-PCR analysis**

| Gene | Primer (5’→3’) |
| --- | --- |
| β-actin | F: CTCATGAAGATCCTGACCGAG |
|  | R: AGTCTAGAGCAACATAGCACAG |
| IL-1β | F: GAAATGCCACCTTTTGACAGTG |
|  | R: TGGATGCTCTCATCAGGACAG |
| IL-6 | F: CCACTTCACAAGTCGGAGGCTTA |
|  | R: AGTGCATCATCGTTGTTCATAC |
| TNF-α | F: AAGGCCGGGGTGTCCTGGAG |
|  | R: AGGCCAGGTGGGGACAGCTC |
| IL-10 | F: GCTCTTACTGACTGGCATGAG |
|  | R: CGCAGCTCTAGGAGCATGTG |
| iNOS | F: CCCTTCCGAAGTTTCTGGCAGCAGC |
|  | R: GGCTGTCAGAGCCTCGTGGCTTTG |
| Arg-1 | F: CTGGGGATTGGCAAGGTGAT |
|  | R: CGTTGAGTTCCGAAGCAAGC |
| Fizz-1 | F: CCAATCCAGCTAACTATCCCTCC |
|  | R: CCAGTCAACGAGTAAGCACAG |
| YM-1 | F: GCAAGACTTGCGTGACTATGAA |
|  | R: AACGGGGCAGGTCCAAA |
| TGF-β | F: ACGTCACTGGAGTTGTACGG |
|  | R: GGGGCTGATCCCGTTGATT |
| mtDNA region 1 | F: TGAACGGCTAAACGAGGGTC |
|  | R: AGCTCCATAGGGTCTTCTCGT |
| mtDNA region 2 | F: CAGTCCCCTCCCTAGGACTT |
|  | R: ACCCTGGTCGGTTTGATGTT |
| mtDNA region 3 | F: TAATCGCACATGGCCTCACA |
|  | R: GAAGTCCTCGGGCCATGATT |
| Ndufa4 | F: CTGGAGCAGCACTGTATGTGA |
|  | R: TTGGGACCCAGTTTGTTCCAT |
| Ndufa8 | F: GAGTTTATGCTGTGCCGCTG |
|  | R: TACTCTGTGAAAGGCTCCGC |
| Ndufa11 | F: TCCGCTTACAGCGTCTCAC |
|  | R: AGGCCAAACATCGCTCCAAT |
| Ndufb3 | F: GAGTTTATGCTGTGCCGCTG |
|  | R: TACTCTGTGAAAGGCTCCGC |
| Ndufb7 | F: GACCCCGAGAAGATACCCAG |
|  | R: GCACAGTAGTCACGTTGCTG |
| Ndufb9 | F: ACCGGTACTTTGCTTGCTTG |
|  | R: ATCTCTCGAAGGAAGTGCCC |
| Ndufb11 | F: GTCCTCCAGGGCTGTAATCG |
|  | R: AAAGTCAGGGTTCTTCGCGT |
| Ndufv1 | F: TGCTTGTGGCTCCGACTATG |
|  | R: ACAGTTGTGGGGCATCCAAA |
| Ndufv2 | F: GGAGGAGCCTTATTTGTGCAT |
|  | R: TTTGGGCGAGATCCAGGACT |
| sdhb | F: CAGAGTCGGCCTGCAGTTT |
|  | R: ATCCAACACCATAGGTCCGC |
| sdhd | F: CTGGTTCCAAGGCTGCATCT |
|  | R: AGCCAGAGAGTAGTCCACCA |
| Cyc1 | F: ATCGTTCGAGCTAGGCATGG |
|  | R: GCCGGGAAAGTAAGGGTTGA |
| Uqcr11 | F: GGAACTGGCCAGAAACTGGA |
|  | R: TGCCGTTGATGTAAGGCACC |
| Uqcrc1 | F: ATGCTGCGTGACATTTGCTC |
|  | R: TAGAAGCGCAGCCAGAACAT |
| Uqcrq | F: ATCTCCTACAGCTTGTCGCC |
|  | R: CTGCTCAAACTCCTGGTTGC |
| Cox5a | F: TGTCTGTTCCATTCGCTGCT |
|  | R: AACCGTCTACATGCTCGCAA |
| Cox5b | F: GCTTCAAGGTTACTTCGCGG |
|  | R: ATGGGTCCAGTCCCTTCTGT |
| Cox6a1 | F: CAACGTGTTCCTCAAGTCGC |
|  | R: CTTCATAGCCGGTCGGAAGT |
| Cox6b1 | F: AGAACTACAAAACTGCCCCCT |
|  | R: TTCTCACAGCGGTGGAAGTC |
| Cox7c | F: GAGTATCCGGAGGTTCACGAC |
|  | R: ACCGCCACTTGTTTTCCACT |
| Cox8a | F: CAGGTCCACTCGAAGCCG |
|  | R: CAGGCAGAAGACAACACACG |
| Cox15 | F: GCGTCCGGCAACGGT |
|  | R: TGATGGTGCTGTACTGTCCT |
| Atp5d | F: TACGCTGACTGGAGCCTTTG |
|  | R: GTCCAGCATGTCCAGTGTCA |
| Atp5e | F: TCAGCTACATCCGGTTTTCCC |
|  | R: TTTTATGCTGCTGCCCGAAG |
| Atp5g2 | F: ATGTACGCCTGCTCCAAGTT |
|  | R: CTGTGGTCGCTTCAACTCCA |
| Atp6v1 | F: ACATCGCAGAGATGGTTCGG |
|  | R: CTTTGGCTGCATCGTAGGGA |
| Atp6v0c | F: GTCCCGTTGTCCTAGCTCGC |
|  | R: TCCTAGAAGCTGGGTGCAGAA |
| Atp5h | F: TGGAATGAGACCTTCCACGC |
|  | R: GCACAGGAATCTTCAGGGCA |
| Atp5k | F: TACCTAAAACCCCGGGCAGA |
|  | R: CATCTTGAGCTTCCGCCAGT |
| NP | F: TGCTTCAAAACAGCCAAGTG |
|  | R: GATGCCCTCTGTTGATTGGT |
| HA | F: GCTGCAGATGCAGACACAAT |
|  | R: CCCTCAGCTCCTCATAGTCG |
